# Restart uncertainty relation for monitored quantum dynamics

**DOI:** 10.1073/pnas.2402912121

**Published:** 2025-01-02

**Authors:** Ruoyu Yin, Qingyuan Wang, Sabine Tornow, Eli Barkai

**Affiliations:** ^a^Department of Physics, Institute of Nanotechnology and Advanced Materials, Bar-Ilan University, Ramat-Gan 52900, Israel; ^b^Department of Computer Science, Research Institute CODE (Cyber Defence), University of the Bundeswehr Munich, Munich 81739, Germany

**Keywords:** uncertainty relation, restart mechanism, monitored quantum dynamics, quantum hitting time

## Abstract

We introduce a time-energy uncertainty relation within the context of restarts in monitored quantum dynamics. Previous studies have established that the mean recurrence time, which represents the time taken to return to the initial state, is quantized as an integer multiple of the sampling time, displaying pointwise discontinuous transitions at resonances. Our findings demonstrate that the natural utilization of the restart mechanism in laboratory experiments, driven by finite data collection time spans, leads to a broadening effect on the transitions of the mean recurrence time. Our proposed uncertainty relation captures the underlying essence of these phenomena, by connecting the broadening of the mean hitting time near resonances, to the intrinsic energies of the quantum system and to the fluctuations of recurrence time. Our uncertainty relation has also been validated through remote experiments conducted on an International Business Machines Corporation (IBM) quantum computer. This work not only contributes to our understanding of fundamental aspects related to quantum measurements and dynamics, but also offers practical insights for the design of efficient quantum algorithms with mid-circuit measurements.

## Significance

A time-energy uncertainty relation is proposed, connecting the fluctuating quantum recurrence time to the energies of the quantum system. This relation reveals a broadening of transitions in the mean quantum recurrence time, which is defined with repeated measurements, and exhibits quantization due to topological effects. Our findings have also been implemented on an IBM quantum computer, further verifying that the uncertainty relation is a consequence of the interplay between unitary dynamics and measurements, rather than noises and decoherence. Broader impacts stemming from these findings are also discussed. The time-energy relation studied here exploits the randomness of the time to complete a monitored process, and in that sense differs from standard formulations where time is a parameter.

The concept of restarting a process is a ubiquitous phenomenon across various disciplines (1, 2). When faced with a setback in reaching a desired goal, the instinct to restart the process often arises, driven by the hope of achieving better success in subsequent attempts. This notion of restarting, or “resetting,” gives rise to a compelling paradigm in the realm of classical stochastic processes (2–18). Diffusion processes with resets are the best-studied example (2). In this scenario, a particle undergoes random diffusion but, at periodic or random intervals, is brought back to its initial position. Additionally, within this framework, a specific target awaits the particle’s arrival, prompting us to inquire about the time it takes for the particle to reach this target for the first time. This random time, both with and without the restart mechanism, is commonly known as the “first passage time” and has garnered widespread attention (19). In particular, the notion of restarts plays a pivotal role in expediting search processes, making these ideas highly relevant and applicable across diverse fields, including biology (20), computer science (21, 22), animal foraging (23–25), the study of chemical reactions (26–28), and quantum dynamics (29–46), among others.

The concept of restarting processes is of particular importance in the context of repeated mid-circuit measurements performed on quantum computers and more generally in the context of monitored quantum walks (47). In quantum dynamics, the notion of “first hitting time” without restart reveals intriguing and novel features, often intimately connected with topological considerations, resonances, and the concept of dark states (47–70). Typically, these processes are represented using graphs, which can describe the states of various quantum systems, such as single particles or qubit systems. Within this graph, a crucial element is the presence of a target state, often symbolizing the measurement device.

To detect the system at the target state, it might be tempting to perform measurements at infinitesimally short intervals. However, this approach encounters the Zeno effect (71), where frequent strong measurements effectively freeze the system’s dynamics, rendering it undetectable. As a solution, a sequence of measurements is performed at regular intervals of *τ* units of time, allowing the system to evolve unitarily between measurements (47–53). Yet, when implementing this fundamental search process on a quantum computer or any practical device, practical challenges emerge. Over time, due to measurement imperfections or interactions with the environment, quantum effects tend to diminish due to noise and decoherence. In such cases, a common strategy is to restart the process. This issue of finite-time resolution is not exclusive to the quantum realm and is encountered in classical systems as well. What distinguishes the quantum realm is the potential for sharp and discontinuous resonances in mean hitting times, related to quantum revivals (72) and topological effects (see below). Remarkably, as shown below, even when the restart time is significantly longer than the mean first hitting time, the act of restarting can have a profound impact. Our objective is to investigate these phenomena by leveraging an uncertainty relation, which is vastly different from previous ones (73–78).

**Fig. 1.**
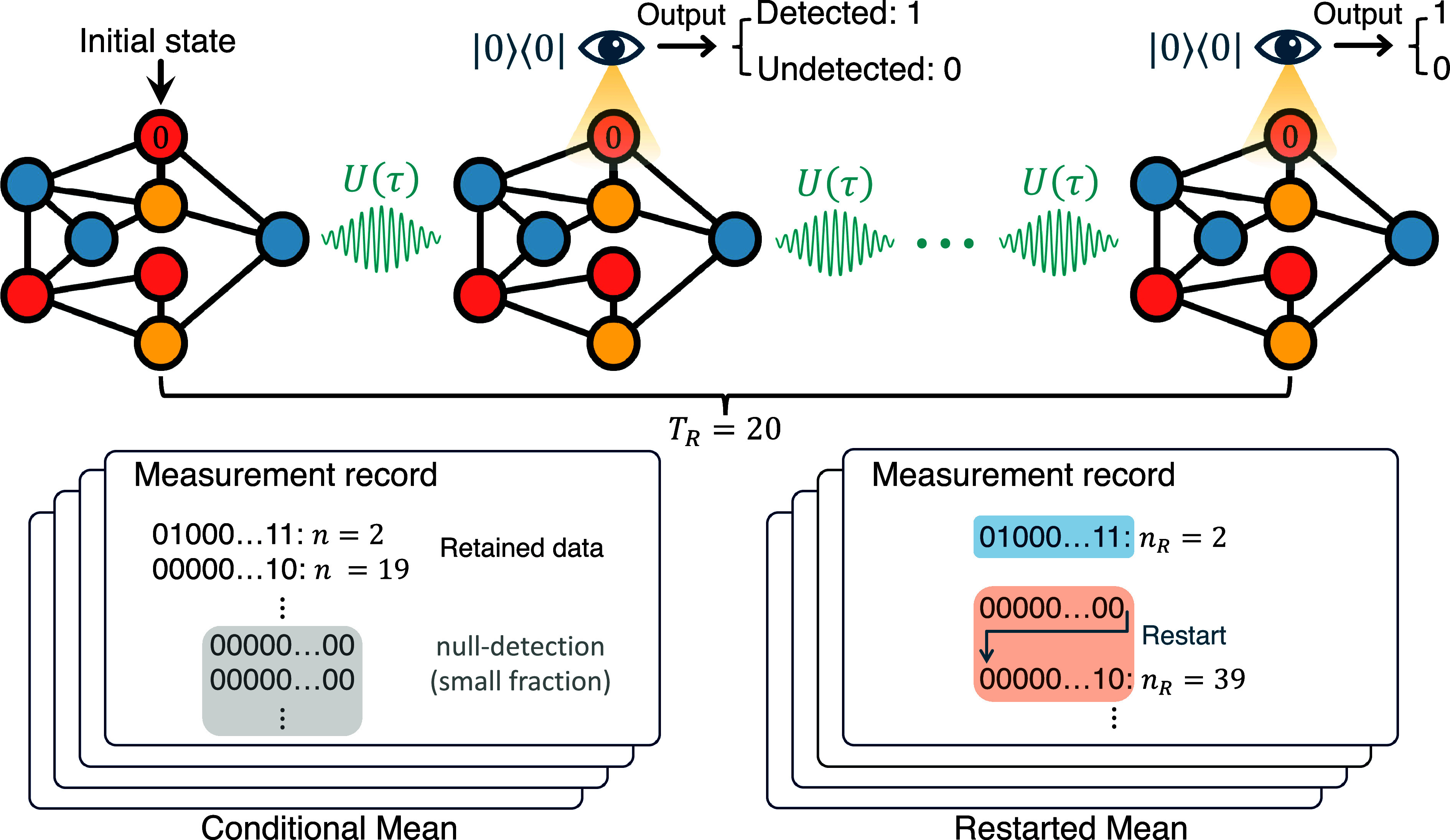
The measurement protocol for monitored quantum walks and its output. A quantum walker on a graph is initialized at the spatial state |0⟩ (marked “0”). A projective measurement at the initial state, schematically presented by the eye symbol, is performed following the unitary evolution of time *τ*. The output of the measurement is either “yes” (1) or “no” (0), rendering the wavefunction of the quantum walker either localized at |0⟩ or its amplitude erased at the state |0⟩. We continue the free evolution immediately after the measurement for another duration *τ*, and then measure again, resulting in another binary outcome: 0 or 1. Using an IBM quantum computer, the process of interrupting evolution by stroboscopic measurements, for a tight-binding three-site ring, was implemented for 20 steps, as a single realization, thus leading to an output string or measurement record of 20 bits. Our goal is to find the number of steps when the first 1 (“yes”) emerges, which is the quantum first hitting time in units of *τ*. Repeating a large number of realizations gives the statistics of hitting times. Two common statistical measures of estimating the mean hitting time are used. In the first, we disregard the (rare) sequences with all 0 measurements, and this yields the mean conditioned on detection. In the second, called restarted hitting time, we continue until the first detection, as illustrated in the figure, leading to the sampling of the mean restarted hitting time. In this example, the restart time is $T_{R}=20$ in units of *τ*.

To illustrate the key aspects of our study, we commence with an experimental demonstration conducted on an IBM quantum computer (https://quantum.ibm.com). In this experiment, we consider a straightforward three-site ring graph with quantum states represented as |0⟩, |1⟩, and |2⟩. The system is described by a tight-binding Hamiltonian that accounts for hopping between these states. Our starting point is state |0⟩, which also serves as the target state for this investigation. We aim to observe the recurrence of the system to its initial state through periodic measurements conducted every *τ* unit of time. The measurement outcomes yield a sequence of “no” responses (indicating null detection) followed by a “yes” response when the target state is eventually detected. The first occurrence of “yes” in this sequence defines the first hitting time (47–53), as demonstrated in Fig. 1. For instance, an experimental outcome might yield the sequence {no,no,yes}, which corresponds to a first detection time of 3τ. Through repeated experiments conducted on the quantum computer, we determine the mean number of measurements required for detection, denoted as ⟨n⟩. This quantity, extracted from the quantum computer, provides us with valuable insight into the average time it takes to detect the target state.

Theoretical investigations, spanning a wide range of graph types, have extensively explored the aforementioned problem (47–62, 66–68). We first present the basic theory ignoring restart, showing that such a theory does not align with the experiments. Notably, Grünbaum and colleagues (47) made a remarkable discovery: The theoretical mean recurrence time exhibits quantization. In practical terms, this implies that the value of ⟨n⟩ is constrained to integer values. Mathematically, this integer is encapsulated by a winding number *w* associated with a generating function and hence the phenomenon is topological. The integer is defined and denoted as

[1] $$\begin{array}{c} \left\langle n\right\rangle =\sum_{n=1}^{\infty }nF_{n}=w. \end{array}$$

Here, *F*_*n*_ is the probability of first detection in the *n*-th measurement, which is normalized, i.e. $\sum_{n=1}^{\infty }F_{n}=1$. It is obtained using the unitary U(τ)= exp(−iHτ) (*ħ* is set as 1, and *H* is the Hamiltonian) describing the evolution between measurements and the projection |0⟩⟨0| describing the measurements using collapse theory, so all along this work |0⟩ is the target state. Specifically (47, 49, 51),

[2] $$\begin{array}{c} F_{n}={\left|\langle 0|U\left(\tau \right)S^{n-1}|0\rangle \right|}^{2}, \end{array}$$

where the survival operator $S=\left(1-|0\rangle \langle 0|\right)U\left(\tau \right)$ (1 is the identity matrix), demonstrating the unitary evolution in the time interval *τ* followed by the complementary projection described by 1−|0⟩⟨0| (indicating null detection). In general, the winding number *w* is computed as follows (47, 55): Given the time-independent Hamiltonian and assuming a finite graph, we search for the energy levels and corresponding states of the system, denoted as $H|E_{k}\rangle =E_{k}\left|E_{k}\right\rangle$. The value of ⟨n⟩=w represents the count of distinct phase factors, such as $e^{-iE_{k}\tau }$, associated with stationary states that exhibit nonzero overlap with the target state. See details including the proof for Eq. **1** in *SI Appendix*.

In our experimental example on the three-site ring (*Model*), we encounter energy level degeneracy, resulting in ⟨n⟩=2 for nearly any choice of *τ*. However, a pivotal observation emerges when the phase factors merge, causing ⟨n⟩ to become equal to 1. The merging of phase factors occurs for specific values of *τ* which are straightforward to identify. Consequently, the relationship between ⟨n⟩ and *τ* is predominantly characterized by the value 2, except for isolated pointwise discontinuities, where it abruptly becomes 1. These peculiar values of *τ* correspond to instances of wave packet revivals, wherein certain times lead to the complete revival of the wave packet to its initial state. During such moments, the first measurement invariably yields a “yes” outcome. What makes this phenomenon particularly extraordinary is the discontinuous nature of ⟨n⟩ and its intriguing insensitivity to values of *τ* beyond the revival times themselves.

**Fig. 2.**
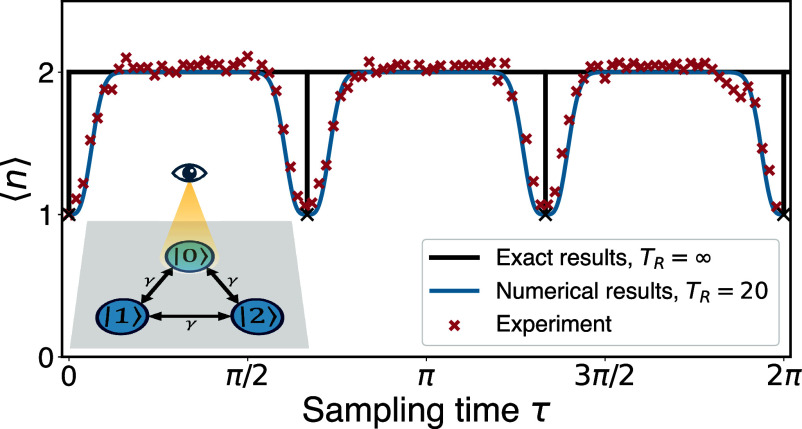
Mean hitting time for the three-site ring model. The numerically/experimentally obtained mean quantum first return time of the three-site ring model. The exact results for $T_{R}=\infty$ (black line), as stated under Eq. **1**, present discontinuous jumps or dips of ⟨n⟩=w, from *w* = 2 to *w* = 1, at τ=2πk/3 (k=0,1,2,⋯). In the experimental data (red crosses, $T_{R}=20$), these transitions are widened. The numerical results for $T_{R}=20$ (blue line) perfectly match the experimental results. In the paper, we address the broadening effect showing how it is related to an uncertainty relation. *Inset* is the scheme of the tight-binding model for a ring with three sites, and *γ* (set as 1) denotes the strength of the hopping matrix element, see Eq. **16**. We measure periodically the target state |0⟩ (indicated with an eye). See details of the IBM remote experiments in *Materials and Methods* and *SI Appendix*.

The theoretical findings described above are valid in principle for infinitely long time measurements, and they have been graphically represented in Fig. 2, alongside the corresponding experimental results from an IBM Eagle processor (IBM Sherbrooke). Notably, the delta-like narrow transitions predicted by the theory are observed to exhibit widening in the real-world experimental data. Nonetheless, a clear alignment between theory and experiment persists, except in the immediate vicinity of these transitions. Importantly, the above-mentioned resonances and broadening effect is a generic phenomenon of first hitting time statistics, and is not limited to the example under study.

The inception of this research stemmed from the natural inquiry: Is this widening phenomenon a generic occurrence? Is it primarily attributed to inherent noise inherent to the system, such as imperfect timing in measurements or the unitary itself or is it potentially linked to the fundamental principles of quantum measurement theory? Specifically, can the basic postulates of quantum measurement theory provide a quantitative description of these transitions? When we refer to a “transition,” or a “topological transition” or “resonance,” we mean the shift of ⟨n⟩=w (as illustrated by *w* = 2 in Fig. 2) to ⟨n⟩=w−1 and back, as we systematically vary the parameter *τ*. In this context, *τ* serves as our control parameter, although it is worth noting that other parameters of the system Hamiltonian could be employed for a similar investigation. We claim below that the widening effects seen in Fig. 2, are generic and are due to the restart paradigm. Second, we find that the widening effects are determined by the fluctuations in the system, or to put it differently, the width of the transition teaches us about the fluctuations of the hitting time. Further, these uncertainties in hitting times are shown to be related to the energies of the system, thus extending the time-energy uncertainty relation to a case where the time is actually fluctuating.

Using mid-circuit measurements, the experimental output typically commences with a sequence of null measurements, characterized by the string {no,no,⋯}. It is important to note that this string is always finite, and its length is denoted as *T*_*R*_ (with the subscript “R” signifying “restart”). In some instances, we encounter a “yes” in the sequence, signifying the successful detection of interest, and thus providing the random hitting time. However, there are cases where we find a sequence composed entirely of “no’s,” implying that no detection has occurred until the time $T_{R}\tau$, see Fig. 1 with $T_{R}=20$. To analyze the statistical features of the experiments, we use basics of restart theory. When we average the results, we focus on two essential statistical measures. The first is the mean, conditioned on detection within the first *T*_*R*_ attempts, denoted as ${\left\langle n\right\rangle }_{\mathrm{Con}}$, is given by:

[3] $$\begin{array}{c} {\left\langle n\right\rangle }_{\mathrm{Con}}=\frac{\sum_{n=1}^{T_{R}}nF_{n}}{P_{\det}}, \end{array}$$

where $P_{\det}:=\sum_{n=1}^{T_{R}}F_{n}$ is defined as the detection probability within time *T*_*R*_. In the estimation of this mean, we exclude all sequences that contain *T*_*R*_ null measurements. The second statistical measure is the restarted mean, which counts all sequences, including those without any “yes,” denoted as $\left\langle n_{R}\right\rangle$. Namely, *n*_*R*_ gives the total number of attempts until the first “yes,” regardless of how many restarts have happened. See the schematics in Fig. 1. Its mean is quantified as (38, 79):

[4] $$\begin{array}{c} \begin{array}{c} \left\langle n_{R}\right\rangle ={\left\langle n\right\rangle }_{\mathrm{Con}}+T_{R}\frac{1-P_{\det}}{P_{\det}}. \end{array} \end{array}$$

The first term on the right-hand side corresponds to paths where detection occurred within *T*_*R*_ attempts, while the second term encompasses paths where detection happened after *T*_*R*_ attempts. Therefore, the mean restart time, $\langle n_{R}\rangle \tau$, provides an estimate of the average time until the first detection, considering an ensemble that does not exclude any specific path. In theory, as *T*_*R*_ tends toward infinity, we obtain the idealized limit as expressed in Eq. **1** from Eqs. **3** and **4**, though precisely in the vicinity of resonances, this limit must be considered with care.

We introduce the variance of detection times, measured in units of *τ*, as:

[5] $$\begin{array}{c} \sigma_{n}^{2}=\left\langle n^{2}\right\rangle -{\left\langle n\right\rangle }^{2}=\sum_{n=1}^{\infty }n^{2}F_{n}-w^{2}. \end{array}$$

This variance, denoted as $\sigma_{n}^{2}$, quantifies the uncertainty associated with the first hitting time. Importantly, this uncertainty tends to be substantial in the proximity of the topological transition under investigation, and notably, these fluctuations become more pronounced as we approach the transition (55). Our main results are relationships between this uncertainty and the restarted process using the following expressions:

[6] $$\begin{array}{c} {\left\langle n\right\rangle }_{\mathrm{Con}}=w-\left(\frac{2T_{R}}{\sigma_{n}^{2}}+1\right)\;\exp\left(-\frac{2T_{R}}{\sigma_{n}^{2}}\right), \end{array}$$

[7] $$\begin{array}{c} \left\langle n_{R}\right\rangle =w-\;\exp\left(-\frac{2T_{R}}{\sigma_{n}^{2}}\right). \end{array}$$

These equations hold in the limit of large *T*_*R*_ and large $\sigma_{n}^{2}$ while keeping the ratio $T_{R}/\sigma_{n}^{2}$ constant. These relationships are general in nature, describing transitions from *w* to *w* − 1, a phenomenon found in a broad class of Hamiltonians when a pair of phase factors merge. When $T_{R}/\sigma_{n}^{2}\to \infty$, signifying a state far from resonance, we observe that ${\left\langle n\right\rangle }_{\mathrm{Con}}=\left\langle n_{R}\right\rangle =w$. Conversely, when $T_{R}/\sigma_{n}^{2}\to 0$, indicating resonance, we find that ${\left\langle n\right\rangle }_{\mathrm{Con}}=\left\langle n_{R}\right\rangle =w-1$. Thus, Eqs. **6** and **7** describe the broadening of the transitions that diminishes as we increase the resetting time. These findings are significant as many aspects of the process, such as the complete spectrum of S or *U*, are unimportant and do not impact the overall outcome. We will soon show that this is related to a type of time-energy relation.

## Experimental Validation

**Fig. 3.**
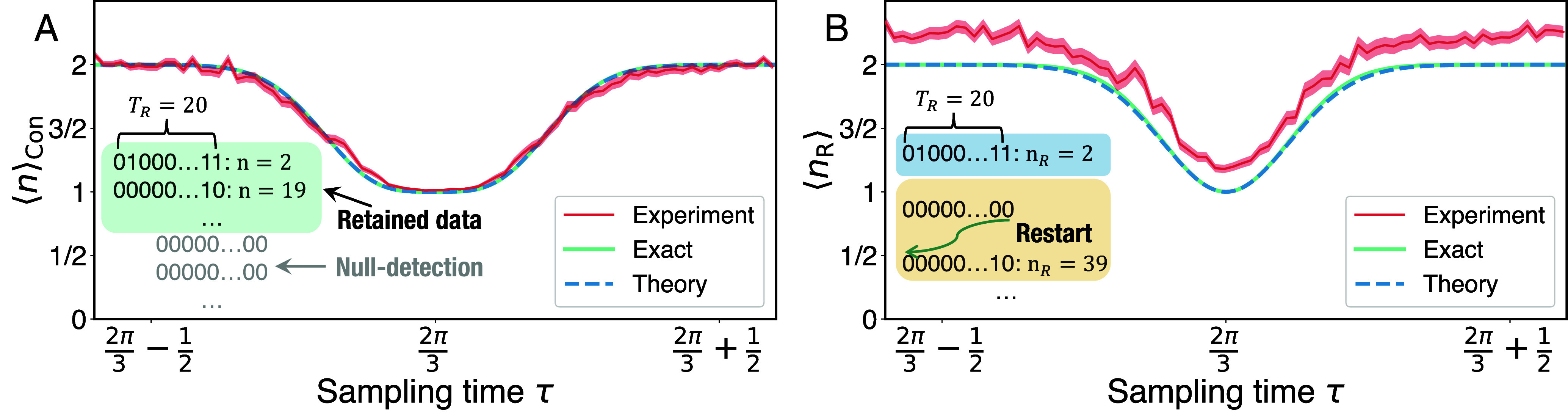
Impact of restart on hitting time transitions. (*A*) The transition from ${\left\langle n\right\rangle }_{\;\text{Con}}=2$ to ${\left\langle n\right\rangle }_{\;\text{Con}}=1$ and back is widened due to restarts. In particular, here we restart after $T_{R}=20$ measurements, as highlighted in the *Insets*. We compare the exact results (green solid line) found using Eqs. **2** and **3** with the theory (blue dashed line) obtained using Eq. **6** and IBM quantum computer experiments (red line). The results clearly demonstrate that basic postulates of measurement theory and the uncertainty relation using the variance of the hitting time perfectly align with the experiment. In turn, noise and imperfect measurements are not factors in the observed behavior. (*B*) The mean hitting time under restart, $\left\langle n_{R}\right\rangle$, as a function of *τ*. We compare the exact results (green solid line), the theory (blue dashed line, computed with Eq. **7**), and experiment results on the IBM quantum computer (red line) for $T_{R}=20$. We observe the vertical shift between the experimental and exact results, which is due to noise in quantum computers, and more specifically, due to a small 1% shift in the detection probability which is discussed in the text. The model here is a tight-binding three-site ring, the same as in Fig. 2. In both figures, the exact results are obtained using Eq. **2**, from which we find *F*_*n*_, and then using Eq. **3** for (*A*) or Eq. **4** for (*B*). The shaded red region represents the CI 99.7%, signifying an interval spanning three SDs above and below the mean in a standard normal distribution.

In the analysis of the experimental data depicted in Fig. 3*A*, we relied on the use of the conditional mean, as described earlier. Additionally, we provided a theoretical representation based on Eq. **6**, which exhibits a remarkable alignment with the experimental results without requiring any fitting procedures. This indicates that the uncertainty relation, solely based on measurement postulates and not noise in the IBM quantum computer, is responsible for the broadening. For these experiments, we set $T_{R}=20$. Interestingly, in Fig. 3*B*, for the restarted mean, we also observe an alignment of the theory with experiment, though now we see a small constant shift between predictions and the data. We now explain this effect.

Consider *τ* in Fig. 3*B* far from resonance, for instance, at τ=2π/3, the theoretical detection probability within time $T_{R}=20$, $P_{\det}=\sum_{n=1}^{T_{R}}F_{n}$, is approximately equal to 1. However, in our experimental observations, we find that $P_{\det}$ is approximately 0.99, indicating a small but notable deviation between theory and experiment. This slight deviation has a noticeable impact on the expected value of *n*_*R*_. Recall that $\left(1-P_{\det}\right)T_{R}/P_{\det}$, i.e. the second term in Eq. **4**, is approximately 0, since $P_{\det}≃1$. However, when we use the experimental values just mentioned, we find that for $T_{R}=20$, $T_{R}\left(1-P_{\;\text{det}}\right)/P_{\;\text{det}}=0.2$. Remarkably, this observed value corresponds exactly to the shift we observe in $\left\langle n_{R}\right\rangle$, as presented in Fig. 3*B* (please refer to *SI Appendix*, Supplementary Note 1 for an in-depth discussion on this issue). We conclude that the small shift is consistent with very small errors in the estimation of the detection probability $P_{\;\text{det}}$.

This situation highlights a crucial point: When *T*_*R*_ is large, even small errors on the order of 1% can result in a visible shift in the experimental outcome, $\left\langle n_{R}\right\rangle$, and this shift grows linearly with *T*_*R*_. A similar effect is not found for the conditional mean. As mentioned, the latter neglects experimental realizations with no detection at all. The conditional mean consistently falls below the restarted mean, a trend particularly noteworthy in search contexts, where the primary objective is to expedite the process. Hence, one should wonder which measure holds greater merit. We believe that both are valuable statistical measures, and there is no point in highlighting one over the other. We will later address the issue of error and noise in our experiment, now we return to the theoretical analysis of the uncertainty relation.

## Uncertainty and Energy

Given that the merging energy phase factors, denoted $\exp\;(-iE_{+}\tau )$ and $\exp\;(-iE_{-}\tau )$, are responsible for the resonances observed, we aim to establish a connection between the restarted and conditional means and the underlying energies within the system. To accomplish this, we provide a sketch of the proof of the main results and extend them. In the limit of a large number of attempts (denoted as *n*), the probability of detection in the *n*-th attempt exhibits exponential decay, as expressed by

[8] $$\begin{array}{c} F_{n}∼a\left(\zeta_{\text{max}}\right){\left|\zeta_{\text{max}}\right|}^{2n}. \end{array}$$

$|\zeta_{\text{max}}|$ is the largest eigenvalue of the survival operator S satisfying $|\zeta_{\text{max}}|<1$. $a(\zeta_{\;\text{max}})$ is a coefficient independent of *n* (which will soon be discussed). A critical aspect to consider is that when we precisely tune *τ* to the resonance, $|\zeta_{\text{max}}|\to 1$ (see below for graphic explanation) (47, 55, 56, 64). As we soon explain at resonance $\lim_{|\zeta_{\;\text{max}}|\to 1}a\left(\zeta_{\;\text{max}}\right)=0$. This occurrence effectively reduces the dimension of the Hilbert space, and this reduction can be demonstrated as the reason for the transition from *w* to *w* − 1 (47), which, in turn, translates to the resonance observed in the hitting time. To gain insight, let us consider a scenario in which two phase factors have exactly merged, specifically when $\exp\;\left(-iE_{-}\tau \right)=\exp\;\left(-iE_{+}\tau \right)$ for some pair of energy levels. In this case, the following state is called dark (56, 64):

[9] $$\begin{array}{c} \left|D\right\rangle =N\left[\left\langle E_{+}|0\right\rangle |E_{-}\rangle -\left\langle E_{-}|0\right\rangle \left|E_{+}\right\rangle \right]. \end{array}$$

Here, *N* is for normalization, and $S|D\rangle =e^{-iE_{+}\tau }\left|D\right\rangle$, indicating that the eigenvalue of S resides on the unit circle. Since this state is orthogonal to the target state |0⟩ and also an eigenstate of the unitary, if we initially populate this state, it is never detected, so it is a dark state. Hence, in our problem, when we adjust the parameter *τ*, which is the focus of our resonance and broadening study, we find that it is intricately linked to the creation of a dark state within the Hilbert space. Further, when the parameters are set close to resonance, $|\zeta_{\;\text{max}}|$ is close to unity, indicating a very slow relaxation of *F*_*n*_, which in turn is responsible for the novel effects of the restarted process.

To continue consider the sum in the numerator of Eq. **3** using Eqs. **1** and **8**

[10] $$\begin{array}{l} \sum_{n=1}^{T_{R}}nF_{n}=w-\sum_{T_{R}}^{\infty }nF_{n}\\ \;\;∼w-a(\zeta_{\;\text{max}})\frac{T_{R}\left(1-|\zeta_{\;\text{max}}{|}^{2}\right)+1}{{\left(1-|\zeta_{\;\text{max}}{|}^{2}\right)}^{2}}{\left|\zeta_{\;\text{max}}\right|}^{2(1+T_{R})}, \end{array}$$

where we summed an infinite series. As mentioned when phase factors match, the right-hand side of Eq. **10**, based on the theorem in ref. 47, must be *w* − 1, when *T*_*R*_ is large. It then follows that, taking the limit $|\zeta_{\;\text{max}}|\to 1$ before $T_{R}\to \infty$ in Eq. **10**, we find $a\left(\zeta_{\;\text{max}}\right)∼\left(1-\right|\zeta_{\;\text{max}}{{|}^{2})}^{2}$, a result that can be reached with rigorous arguments. Applying a similar procedure to the denominator of Eq. **3** and to Eq. **4** leads to the following main result: Let $\rho =T_{R}\left(1-\right|\zeta_{\;\text{max}}{|}^{2})$, when $|\zeta_{\;\text{max}}|\to 1$ and $T_{R}\to \infty$, we find

[11] $$\begin{array}{c} {\left\langle n\right\rangle }_{\mathrm{Con}}=w-\left(\rho +1\right)e^{-\rho }\;\;\text{and}\;\left\langle n_{R}\right\rangle ={\left\langle n\right\rangle }_{\mathrm{Con}}+\rho e^{-\rho }. \end{array}$$

These formulas relate the resonances and the broadening to both the slowest decaying channel in the problem, i.e. to the eigenvalue $\zeta_{\;\text{max}}$, and the restart time *T*_*R*_. They show how an analysis of the spectrum of the survival operator, in particular, the finding of its largest eigenvalue $|\zeta_{\;\text{max}}|<1$, is crucial for the problem.

We now consider the fluctuations of the hitting time. Splitting the sum Eq. **5** into two, we have

[12] $$\begin{array}{c} \sigma_{n}^{2}=\sum_{n=1}^{k_{c}}{\left(n-w\right)}^{2}F_{n}+\sum_{k_{c}+1}^{\infty }{\left(n-w\right)}^{2}F_{n}. \end{array}$$

Choosing a large value of *k*_*c*_ such that we can use Eq. **8**, summing an infinite series we find (55) $\sigma_{n}^{2}∼2/(1-|\zeta_{\;\text{max}}{|}^{2})$. This quantifies the statement made before: The fluctuations are large close to the transition since $|\zeta_{\;\text{max}}|≃1$. Using this relation between the uncertainty *σ*_*n*_ and the eigenvalue $\zeta_{\;\text{max}}$ we obtain Eqs. **6** and **7**. A rigorous proof, including the validity of Eq. **8**, is provided in *SI Appendix*, Supplementary Note 2.

To complete the physical picture, namely, connect the resonance width with the energies of the system, we use the results in ref. 55. A perturbation theory, where the small parameter is the small arc on the unit disk, connecting the two nearly merging phases $\exp\;(-iE_{-}\tau )$ and $\exp\;(-iE_{+}\tau )$, was used to find $\zeta_{\;\text{max}}$. The results in ref. 55 gives ${\left|\zeta_{\;\text{max}}\right|}^{2}∼1-\lambda {\left(\tilde{\Delta E\tau }\right)}^{2}$ (parameters soon to be defined). Then with Eq. **11** we find

[13] $$\begin{array}{c} {\left\langle n\right\rangle }_{\;\text{Con}}=w-\left[1+\lambda T_{R}{\left(\tilde{\Delta E\tau }\right)}^{2}\right]\exp\;\left[-\lambda T_{R}{\left(\tilde{\Delta E\tau }\right)}^{2}\right], \end{array}$$

[14] $$\begin{array}{c} \left\langle n_{R}\right\rangle =w-\exp\;\left[-\lambda T_{R}{\left(\tilde{\Delta E\tau }\right)}^{2}\right], \end{array}$$

where $\lambda =p_{+}p_{-}/{\left(p_{+}+p_{-}\right)}^{3}$ with the overlaps $p_{\pm }=\sum_{l}^{g_{\pm }}\left|\langle 0\right|E_{\pm ,\;l}\rangle {|}^{2}$ ($g_{\pm }$ is the degeneracy of the energy level $E_{\pm }$), and

[15] $$\begin{array}{c} \tilde{\Delta E\tau }:=\tau \left|E_{+}-E_{-}\right|\;\mathrm{mod}\;2\pi . \end{array}$$

Eqs. **13** and **14** clearly show the dependence of the mean hitting time on the system energies, and also practically, are employed to obtain the theoretical results in Fig. 3. At resonances, when $\tilde{\Delta E\tau }=0$, both ${\left\langle n\right\rangle }_{\;\text{Con}}$ and $\left\langle n_{R}\right\rangle$ are equal to *w* − 1. Additionally, the resonance width decreases when we increase the restart time, assuming all other parameters remain constant.

**Fig. 4.**
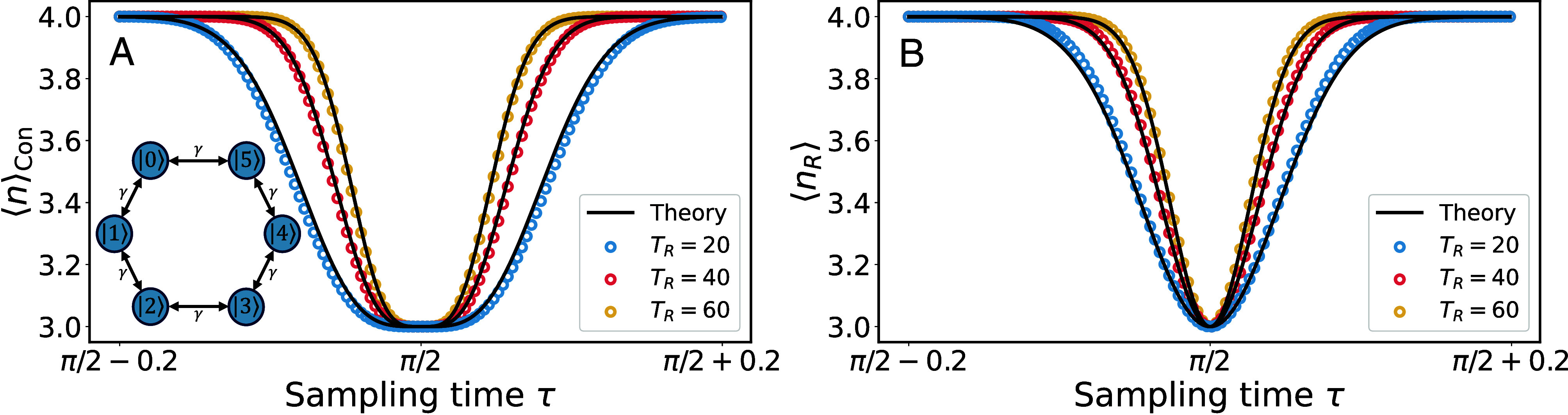
The Broadening of the Hitting Time Transitions in the Benzene-Type Ring Model. (*A*) The conditional mean ${\left\langle n\right\rangle }_{\;\text{Con}}$ and (*B*) the restart mean $\left\langle n_{R}\right\rangle$ as a function of *τ*. The model here is the benzene-type ring (Eq. **16** with *L* = 6 and *γ* = 1), and we work in the vicinity of its critical sampling time τ=π/2, with the transition ⟨n⟩=4 to ⟨n⟩=3. The black lines represent the theory from Eqs. **6** and **7**. The dots represent the numerical exact results obtained using Eq. **2**. In the figures, from the *Bottom* to the *Top* line, the restart time *T*_*R*_ is 20, 40, and 60, respectively. Clearly, the transition is narrowed when *T*_*R*_ grows. *Inset* is the scheme of the benzene-type ring model, and the target state is |0⟩.

**Fig. 5.**
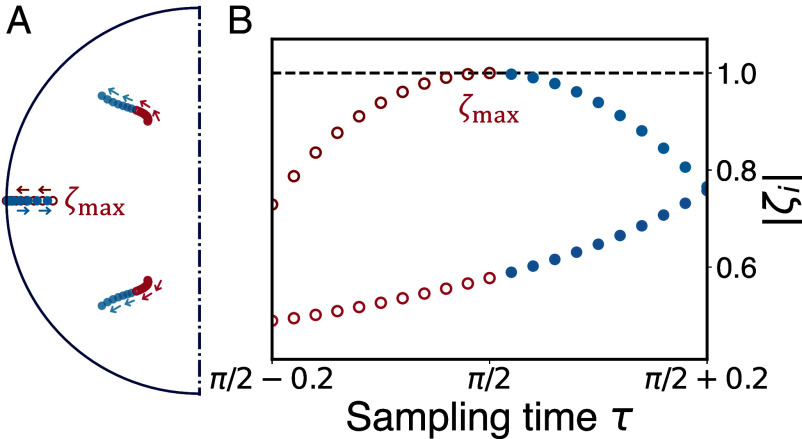
Eigenvalue analysis in the benzene-type ring model. The eigenvalues $\left\{\zeta_{i}\right\}$ of the survival operator S for the six-site ring model, with the sampling time *τ* varied in the same range as in Fig. 4. Recall that $\left|\zeta_{i}\right|$ in general are less or equal unity. In (*A*) we present the eigenvalues as the sampling time *τ* is varied, and the semicircle is of radius 1. In (*B*) we plot the absolute values of $\left\{\zeta_{i}\right\}$. Due to the degeneracies of S, we have three eigenvalues. As shown in (*A*), two eigenvalues (conjugate to each other) are far away from the unit circle and hence become irrelevant. One eigenvalue approaches the unit circle, and is solely responsible for the hitting time statistics and the uncertainty relation. We use arrows to illustrate entering or exiting the resonance at τ=π/2. The red open circles present the eigenvalues when entering the resonance, and blue closed circles are used for the ones when exiting the resonance. The corresponding behaviors of the distance of the eigenvalues $\left\{\zeta_{i}\right\}$ to the origin are demonstrated in (*B*), where the two irrelevant eigenvalues share one set of data presented by the lower circles. Clearly, we see $|\zeta_{\;\text{max}}|$ goes to 1 and back when entering and exiting the resonance. As explained in the text, when $|\zeta_{\;\text{max}}|=1$ we have a dark state in the system; see Eq. **9**.

We tested our theory using several model systems. For example, a benzene-type ring (Eq. **16** with *L* = 6), as presented in Fig. 4, where excellent agreement between the theory and numerically exact results is witnessed. We see, as predicted by Eqs. **13** and **14**, the width of the transition becomes smaller as the restart time *T*_*R*_ grows. See details of other graph models in *SI Appendix*. To verify the uniqueness of $\zeta_{\;\text{max}}$, in Fig. 5, we present the behaviors of the eigenvalues $\left\{\zeta_{i}\right\}$ for the model of benzene-type ring, when the sampling time *τ* is varied. One of the eigenvalues, namely $\zeta_{\;\text{max}}$, approaches the unit circle when *τ* goes to π/2, while the other pair of conjugate eigenvalues are relatively far from the unit circle. As previously stated, when the largest eigenvalue $|\zeta_{\;\text{max}}|$ approaches the unit disk, the relevance of the other eigenvalues is negligible and the restart uncertainty relation presented in this work becomes relevant.

A natural query is to study the effects of system size on our main results. To this aim, we analyzed two models: the ring model and the complete graph with *L* sites. The case *L* = 3 corresponds to the experimental study we conducted. For *L* > 3, the results exhibit distinct behaviors. Focusing on the merging of two phases, corresponding to the largest and ground state energy, we find w=1+L/2 (w=(1+L)/2) for the even (odd) ring model and *w* = 2 for the complete graph. Assuming the hopping amplitude *γ* (as indicated in the *Inset* of Fig. 2 and Eq. **16**) is *L*-independent, the width of the resonance decreases as we increase *L* (*SI Appendix*, Supplementary Note 3). However, considering the resonance related to the first excited state and the ground state, for the ring model we find that the resonance width will increase as the size of the system grows. The complete graph has merely two energy levels hence this choice of energy levels is clearly the same as the min-max choice, mentioned above. The key issue for the broadening effect is how the energy gaps and the parameter *λ* in Eqs. **13** and **14** scale with the size of the system. *w* depends on the symmetry of the system and the degeneracy of the energy levels. For example, in the complete graph, the number of distinct energy levels is two for any *L*, which means *w* = 2. This results in relatively short mean hitting times in units of *τ* compared to the ring model. Importantly, these different behaviors are all captured by our time-energy-like restart uncertainty principle.

## Effect of Random Perturbations

The broadening of resonances in the first hitting time can arise from various sources. In the triangle model implemented on the IBM quantum computer, we have demonstrated that this broadening is attributable to the foundational principles of quantum theory and the restart paradigm. However, a broader objective is to explore the relationship between stochastic perturbations and these broadening effects, and to determine whether the observed topological invariant *w* is resilient to fluctuations of parameters. This investigation, whose details are provided in *SI Appendix*, Supplementary Note 4, encompasses fluctuating sampling times, as well as randomness in restart times.

**Fig. 6.**
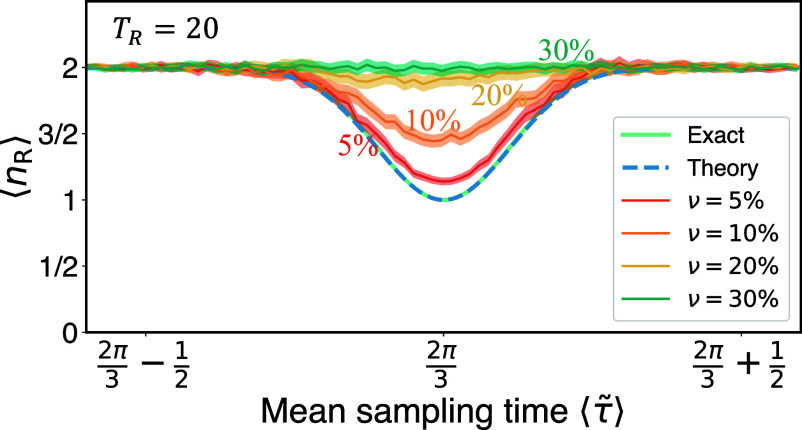
Mean hitting time versus the mean sampling time $\left\langle \tilde{\tau }\right\rangle$, for the three-site ring model with varying fluctuation levels in the evolution time *τ* and fixed $T_{R}=20$. Utilizing the Monte Carlo method with 30,000 realizations, we find that as the fluctuations of *τ* increase, the resonances are progressively diminished, yet the topological number $\langle n_{R}\rangle =2$, far from the resonance, remains unaffected and exhibits robustness. The shaded region represents the CI 99.7% as in Fig. 3.

Utilizing the three-site ring model, we studied the effect of random sampling time and random restart time on our key results. Using $T_{R}=20$, as we did in the experiment, allowing for fluctuations of up to five percent in the sampling time *τ* did not alter our main conclusions. However, when fluctuations in the sampling time *τ* reached 30 percent, the dip in the resonances became difficult to observe, as shown in Fig. 6. There $\tilde{\tau }$ is the actual sampling time, uniformly distributed on [τ(1−ν),τ(1+ν)], and *ν* indicates the fluctuation level. In addition, we found that the resonance is diminishing when *T*_*R*_ is increased, for a fixed fluctuation level of *τ* (*SI Appendix*, Fig. S15). Thus, the larger *T*_*R*_ is, the more pronounced the effects of random sampling times are. Interestingly, the topological invariant far from the resonance, $\langle n_{R}\rangle ≃w=2$, remained robust even with significant fluctuations and large *T*_*R*_, indicating the resilience of this number (Fig. 6 and *SI Appendix*, Fig. S15). Similar behaviors are also observed for the benzene-type ring model, see *SI Appendix*, Fig. S14.

To study the effects of random restart time *T*_*R*_, we focused on two models, assuming $\langle T_{R}\rangle =20$, motivated by our experiments. Using a narrow distribution of *T*_*R*_ (a tent-like distribution) and a model where *T*_*R*_ is Poisson distributed (a relatively wide distribution), we show in *SI Appendix* that the effects of random *T*_*R*_ are marginal (*SI Appendix*, Figs. S16 and S17). This is because of two reasons: The location of resonances is insensitive to *T*_*R*_, as they are controlled by energies and the sampling time and because we use (roughly) symmetric around the mean distributions for *T*_*R*_. It should be noted that the restart mechanism is a classical process, though one could extend it to consider a quantum coin-tossing process for the restart itself. In *SI Appendix*, we outline the Pal-Reuveni framework (6) for random and discrete restart times, suitable for our study.

Our findings show that the restart time-energy uncertainty relation does not change considerably for the restart time distributed symmetrically about its mean, compared with the fixed restart time theory. And this type of resilience also remains when the stroboscopicity of our measurement protocol is perturbed (fluctuating *τ*) and when the measurement time *T*_*R*_ is not vastly exceeding 40 (for the fluctuation level *ν* = 5% which is already exaggerated on current-day quantum computers). Notably, the topological number far from resonance is robust to both significant fluctuations of *τ*, and long measurement time *T*_*R*_. Although the fluctuations in *T*_*R*_ are not likely to happen in current quantum computing platforms, we speculate that nonprecise sampling times are not rare and might stem from noise and errors on quantum computers, suggesting a wider range of applications of the restart uncertainty relation on noisy quantum simulation and computations.

## Impact of Quantum Error and Noise

**Fig. 7.**
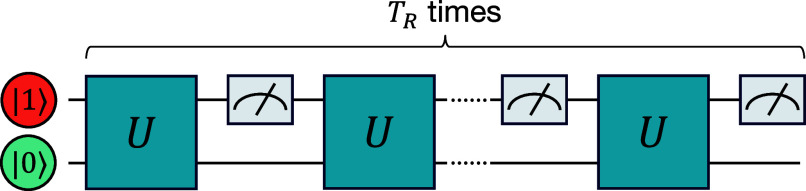
Quantum circuit representation for the three-site ring model. Quantum circuit for two qubits representing the three localized states with alternating unitary *U* and measurements, with the initial state and target state |0⟩=|01⟩.

We now return to the issue of quantum error and noise existing in our experimental implementation. Note that in our IBM experiments, we used two qubits; see *Materials and Methods*. This means that we have four states: |01⟩, |00⟩, |10⟩, and |11⟩, where |11⟩ is theoretically decoupled from the other three while the first three states correspond to the graph states |0⟩,|1⟩,|2⟩, respectively. By measuring the second qubit, we determined whether the system was in the target state |01⟩. Ideally, the operations should isolate the system from |11⟩, but noise existing on the quantum processors causes minor leakage into this state, rendering the deviations in $P_{\det}$ as mentioned, and affecting the restart recurrence time. A key issue is to develop noise models that accurately capture the shift observed in Fig. 3*B*, necessitating a detailed analysis of the quantum circuit under consideration. Incorporating IBM-provided noise models (*SI Appendix* and ref. 80), into the same quantum circuit employed in the experiment (Fig. 7 in *Materials and Methods*), namely a four-state model, we simulated this effect, revealing an upward shift in $\left\langle n_{R}\right\rangle$ (*SI Appendix*, Fig. S3). This is consistent with our experimental findings (Fig. 3*B*). More specifically, we incorporated bit-flip errors and thermal relaxation noise models (*SI Appendix*). A key feature of these models is the transfer of amplitude to the theoretically forbidden state, namely a leakage effect which is captured by the four-state model.

While the error in our experiment is roughly 1%, as mentioned, one might wonder what happens if the noise levels increase. We anticipate a transition of the recurrence time to its classical limit. The relevant classical theory, based on a random walk picture, suggests that for a two-qubit system like the one we used, with four states, we would expect ⟨n⟩=4 according to Kac’s theorem (81) when $T_{R}\to \infty$. In this classical limit, no resonances are observed. This discussion highlights that the quantum hitting times we measured are consistently shorter than this classical limit. Whether a quantum-to-classical transition in the first hitting times occurs due to increased noise levels remains an open question for future work.

## Discussion

In a broader perspective, the observed transitions exhibit similarities to line-shape resonances and broadening encountered across various fields of spectroscopy (82). However, a distinguishing feature here is that the periodic driving force is not an external field acting upon a material system. Rather, they arise from the intrinsic nature of the measurements themselves and their periodicity. Notably, resonances are associated with the creation of dark states, in contrast to traditional resonances linked to quanta of energy carried by particles such as photons. Dark states are commonly observed in quantum systems, often appearing as dips in line shapes due to destructive interference, for example in electromagnetically induced transparency (83–85) and coherent population trapping (86–91) experiments. In the recurrence problems, where we measure the mean hitting time, these states play a unique role. Similar to the role of dark states in other fields, where they enhance effects like laser cooling (92–94), the formation of dark states in our context leads to a speedup of the recurrence time. This acceleration occurs because dark states reduce the effective size of the Hilbert space, making searches more efficient and resulting in faster detection at resonances. This holds true for the recurrence problem, namely the initial condition under study is detected with probability one if $T_{R}\to \infty$, so we are focusing on a bright state all along, though our observable ⟨n⟩ is clearly influenced by the creation of dark states in the Hilbert space.

The broadening of the resonances of recurrence time is intricately linked to three crucial factors: the uncertainty *σ*_*n*_, the slowest decaying mode in the problem, i.e. $|\zeta_{\;\text{max}}|$, and the energies of a pair of merging phase factors. This interconnection establishes fundamental relationships between quantum hitting time statistics and the system’s underlying characteristics, with the restart time playing a pivotal role. It is noteworthy that analogous resonances may be present in related scenarios, particularly when we venture beyond the recurrence problem or engage in nonlocal measurements (63). The expansion of our findings to encompass other observables and the exploration of cases where degeneracies are associated with the absolute value of the eigenvalue $|\zeta_{\;\text{max}}|$, resulting in nonpure exponential decay of *F*_*n*_ and transitions from w→w−2 or w→w−3, etc., rather than the studied w→w−1 case, represents an avenue for future research.

Additionally, we have devised a method for detecting resonances and quantifying their widths in the context of restarted hitting times on quantum computers. We anticipate this to be a valuable tool for investigating the interplay between mid-circuit measurements and unitary operations. The width of the resonance can serve as an indicator of whether the fundamental postulates of measurement theory are effectively functioning on a given device or if noise and decoherence are exerting control. In our experimental study, which was remotely conducted on an IBM quantum computer, we demonstrated that the former scenario holds true. However, we anticipate that, as we increase the size of the quantum system or adjust the restart time, distinct behaviors related to the coupling of these systems to the environment may emerge. Such insights will provide valuable information on the operating conditions of the generation of algorithms with mid-circuit measurements, e.g. dynamic circuits (95) and error correction (96). Furthermore, quantum dynamics driven by measurements has emerged as an intriguing method to study novel phenomena, for example, entanglement transitions (97, 98), induced chirality (99), and synchronization (100). When implemented on a quantum computer, finite-time effects and hence restart will likely emerge as important.

The strategy of restarts used here is nearly mandatory for several reasons. In real quantum circuits, noise and leakage are present. Hence, to study the quantumness of the problem, one is obliged to use finite-time experiments. More generally, unless one finds a way to perfectly correct noise and eliminate leakage in quantum computers with mid-circuit measurements, the restart strategy is nearly a must. The significance of the broadening effect becomes crucial close to discontinuous behaviors of the hitting time statistics, leading to a time-energy uncertainty relation deeply related to the variance of the first detection time. This insight, promisingly, holds the potential to contribute to a better understanding and design of efficient quantum algorithms, which rely on backtracking (restart) and monitored dynamics (101). More importantly, we provided a restart hitting time uncertainty relation, and since hitting times are fluctuating, the uncertainty relation differs from the standard time-energy relation, where time is a parameter and not an observable.

## Materials and Methods

### Model.

The example we considered in the main text is a ring model governed by the nearest-neighbor tight-binding Hamiltonian

[16] $$\begin{array}{c} H=-\gamma \sum_{j=0}^{L-1}\left(|x\rangle \langle x+1|+|x\rangle \langle x-1|\right), \end{array}$$

where *γ* is the hopping amplitude, *L* is the size of the system, and {|x⟩} are the spatial states composing the ring system. As noted, the main results in the manuscript are generally valid and are not limited to this model. The periodical boundary condition indicates |0⟩=|L⟩, and |0⟩ is the target state. The eigenvalues of the Hamiltonian [**16**] are

[17] $$\begin{array}{c} \begin{array}{c} E_{k}=-2\gamma \cos\theta_{k}, \end{array} \end{array}$$

with $\theta_{k}=2\pi k/L$ and k=0,1,2,⋯,L−1. The corresponding eigenstates are $|E_{k}\rangle =\sum_{x=0}^{L-1}e^{i\theta_{k}x}\left|x\right\rangle /\sqrt{L}$. Hence, the overlap is ${\left|\langle x|E_{k}\rangle \right|}^{2}=1/L$. In the main text, for simplicity, we set the hopping amplitude *γ* as 1.

*The three-site ring* was used in our remote IBM experiments. Using Eq. **17** with *L* = 3, there are 2 distinct energy levels, {−2,1}, with $|\left\langle x|E_{k}\right\rangle {|}^{2}=1/3$ and the energy level $E_{1}=E_{2}=1$ is doubly degenerate. Hence, the overlaps are $p_{-}=2/3$, and $p_{+}=1/3$. When τ=2πj/3 with j=0,1,2,⋯, the mean ⟨n⟩ for $T_{R}\to \infty$ jumps from *w* = 2 to *w* = 1, where energy phases $\left\{e^{-i\tau },e^{i2\tau }\right\}$ match. Using the above mentioned $p_{-}$ and $p_{+}$ and energies, Eqs. **13** and **14** give λ=2/9, and $\tilde{\Delta E\tau }=\left|3\tau -2\pi j\right|$ close to each τ=2πj/3. In Fig. 3, *j* = 1 and the resonance condition τ=2π/3 is used. As mentioned, these jumps in the mean hitting time correspond to revivals of the wave packet on the origin.

*The benzene-type ring* was used in our examples plotted in Fig. 4. Here, *L* = 6 and the distinct energies are {±2,±1}, where the energies ±1 are two-fold degenerate. Hence, the overlaps corresponding to distinct energies are $|\left\langle 0|E_{0}=-2\right\rangle {|}^{2}={\left|\left\langle 0|E_{3}=2\right\rangle \right|}^{2}=1/6$, and $|\left\langle 0|E_{1}=-1\right\rangle {|}^{2}+|\left\langle 0|E_{5}=-1\right\rangle {|}^{2}=|\left\langle 0|E_{2}=1\right\rangle {|}^{2}+{\left|\left\langle 0|E_{4}=1\right\rangle \right|}^{2}=1/3$. Using Eq. **1** we therefore expect that, except for a small subset of *τ*’s, ⟨n⟩=4. When τ=(2j+1)π/2 with j=0,1,2,⋯, ⟨n⟩ for $T_{R}\to \infty$ jumps from *w* = 4 to *w* = 3, where the energy phases $\left\{e^{i2\tau },e^{-i2\tau }\right\}$ merge, hence $E_{+}$ and $E_{-}$ used in the text are −2 and 2, respectively. So the parameters in Eqs. **13** and **14** are, λ=3/4, and $\tilde{\Delta E\tau }=\left|4\tau -2\pi (2j+1)\right|$ close to each τ=(2j+1)π/2. In Fig. 4, *j* = 0 or τ=π/2 is used.

### Sketch of the Rigorous Proof for the Uncertainty Relation.

To prove the uncertainty relation, the key is to validate Eq. **8**. Briefly speaking, this can be done via the generating function method (51). Applying the *Z*-transform to the expression inside the bracket of Eq. **2**, i.e. $\phi_{n}=\langle 0|U\left(\tau \right)S^{n-1}\left|0\right\rangle$, one can obtain the generating function, $\tilde{\phi }\left(z\right)=\sum_{n=1}^{\infty }z^{n}\phi_{n}$. Decomposed by the Hamiltonian’s eigenstates, and being a polynomial, $\tilde{\phi }\left(z\right)$ can be factorized by its zeros and poles, using Blaschke factorization (47). Due to the mathematical property of the latter, the poles are the reflection of the zeros, with respect to the unit circle. And also, the zeros are the complex conjugate of the eigenvalues, $\left\{\zeta_{i}\right\}$, of the survival operator S (*SI Appendix*, Supplementary Note 2) (56). Hence, the generating function $\tilde{\phi }\left(z\right)$ can be completely factorized by the zeros, or the eigenvalues $\left\{\zeta_{i}\right\}$. This allows us, in terms of $\left\{\zeta_{i}\right\}$, to use the residue theorem, to recover *ϕ*_*n*_ via the inversion formula $\phi_{n}=\frac{1}{2\pi i}\oint_{|z|=1}\tilde{\phi }\left(z\right)\;z^{-(n+1)}\;dz$. And then $F_{n}={\left|\phi_{n}\right|}^{2}$ can be computed and simplified to Eq. **8**. The detailed derivation is presented in *SI Appendix*, Supplementary Note 2.

### Implementation on a Quantum Computer.

We design a three-site ring model, Fig. 2, using Eq. **16** with *L* = 3. To realize the three-site system on a quantum computer, we use two qubits, which can generate four states: |00⟩,|01⟩,|10⟩ and |11⟩. Hence, we employ the following mapping between the qubits and spatial states representation: |01⟩→|0⟩,|00⟩→|1⟩and|10⟩→|2⟩. We design our circuit in such a way that the additional state |11⟩ is not connected to the others and will never be detected at least theoretically.

In our study, we detect the state |0⟩→|01⟩. This can be realized by measuring only the upper (right) qubit. Hence, when measuring the upper (right) qubit in state |0⟩, the measurement does not give any information to distinguish the state |1⟩→|10⟩ and |2⟩→|00⟩. Importantly, measuring the upper (right) qubit in state |1⟩ tells that the system is in |0⟩→|01⟩ with certainty. We determine the first detection time, *n*, by analyzing mid-circuit measurement outputs from the quantum circuit, as shown in Fig. 7.

We examine the expected value of *n* as a function of *τ*, considering the detection of the target state, namely the upper (right) qubit being detected in state |1⟩, as the endpoint of measurement. As detailed earlier, measurements restart at finite *T*_*R*_, yielding output strings like {0,1,0,1,1,⋯}, of length *T*_*R*_, with “0” and “1” indicating the state of the upper (right) qubit, or actually failure and success in detection, respectively. The experiment ideally concludes after the first appearance of “1,” but due to technological constraints, we cannot terminate the quantum computation based on the measurement outputs, necessitating a finite and constant *T*_*R*_.

The maximum duration for measurement repetitions in the IBM quantum computer IBM Sherbrooke is set at $T_{R}≃20$. This restriction is influenced by software limitations specific to the quantum computer we used. This choice is also chosen to reduce noise and avert nonunitary actions and probability leakage. Such occurrences could render the system’s Hamiltonian (*H*) effectively non-Hermitian. In particular, when performing our experiments on IBM Sherbrooke, $T_{R}=20$ was the maximum number of repeated measurements allowed by the software.

As shown in Fig. 1, to calculate the conditional mean ${\left\langle n\right\rangle }_{\;\text{Con}}$, we disregarded null-detection strings, which are strings of length twenty with only zeros {0,0,⋯,0}. Such strings are rare, since the $P_{\;\text{det}}$ within 20 measurements is nearly 1 (at most 2 percent below 1, depending on *τ*), see details and figure for $P_{\;\text{det}}$ in *SI Appendix*. For the restarted mean, we analyze the unconditional hitting time with restarts, noting the first detection time as *n*_*R*_. For example, consider the sequence of {0,0,⋯,0} of length 20, which, after a restart event, is followed by {0,0,1,⋯}. Here, the first time for detection under restart is $n_{R}=23$. In total, we conducted 32,000 runs with $T_{R}=20$ bits per run, requiring additional data processing to identify the first “1” in each string, thus obtaining the first hitting time *n* for each run. See *SI Appendix*, Supplementary Note 5 for more details on the quantum circuit implementation, error suppression, and data processing.

## Supplementary Material

Appendix 01 (PDF)

## Data Availability

Datasets from experiments data have been deposited in Zenodo (https://doi.org/10.5281/zenodo.13327746) (102).

## References

[1] M. R. Evans, S. N. Majumdar, Diffusion with optimal resetting. J. Phys. A Math. Theor. **44**, 435001 (2011).

[2] M. R. Evans, S. N. Majumdar, Diffusion with stochastic resetting. Phys. Rev. Lett. **106**, 160601 (2011). 21599344 10.1103/PhysRevLett.106.160601

[3] A. Pal, Diffusion in a potential landscape with stochastic resetting. Phys. Rev. E **91**, 012113 (2015). 10.1103/PhysRevE.91.012113 25679576

[4] A. Pal, A. Kundu, M. R. Evans, Diffusion under time-dependent resetting. J. Phys. A Math. Theor. **49**, 225001 (2016).

[5] S. Eule, J. J. Metzger, Non-equilibrium steady states of stochastic processes with intermittent resetting. New J. Phys. **18**, 033006 (2016).

[6] A. Pal, S. Reuveni, First passage under restart. Phys. Rev. Lett. **118**, 030603 (2017). 28157357 10.1103/PhysRevLett.118.030603

[7] S. Belan, Restart could optimize the probability of success in a bernoulli trial. Phys. Rev. Lett. **120**, 080601 (2018). 29543022 10.1103/PhysRevLett.120.080601

[8] M. R. Evans, S. N. Majumdar, Run and tumble particle under resetting: A renewal approach. J. Phys. A Math. Theor. **51**, 475003 (2018).

[9] A. Masó-Puigdellosas, D. Campos, V. Méndez, Anomalous diffusion in random-walks with memory-induced relocations. Front. Phys. **7**, 00112 (2019).

[10] A. Pal, I. Eliazar, S. Reuveni, First passage under restart with branching. Phys. Rev. Lett. **122**, 020602 (2019). 30720306 10.1103/PhysRevLett.122.020602

[11] M. R. Evans, S. N. Majumdar, G. Schehr, Stochastic resetting and applications. J. Phys. A Math. Theor. **53**, 193001 (2020).

[12] O. Tal-Friedman, A. Pal, A. Sekhon, S. Reuveni, Y. Roichman, Experimental realization of diffusion with stochastic resetting. J. Phys. Chem. Lett. **11**, 7350–7355 (2020). 32787296 10.1021/acs.jpclett.0c02122 PMC7586404

[13] B. De Bruyne, J. Randon-Furling, S. Redner, Optimization in first-passage resetting. Phys. Rev. Lett. **125**, 050602 (2020). 32794864 10.1103/PhysRevLett.125.050602

[14] D. Gupta, C. A. Plata, A. Pal, Work fluctuations and jarzynski equality in stochastic resetting. Phys. Rev. Lett. **124**, 110608 (2020). 32242734 10.1103/PhysRevLett.124.110608

[15] S. Gupta, A. M. Jayannavar, Stochastic resetting: A (very) brief review. Front. Phys. **10**, 789097 (2022).

[16] W. Wang, A. G. Cherstvy, R. Metzler, I. M. Sokolov, Restoring ergodicity of stochastically reset anomalous-diffusion processes. Phys. Rev. Res. **4**, 013161 (2022).

[17] B. De Bruyne, S. N. Majumdar, G. Schehr, Optimal resetting brownian bridges via enhanced fluctuations. Phys. Rev. Lett. **128**, 200603 (2022). 35657896 10.1103/PhysRevLett.128.200603

[18] A. Pal, V. Stojkoski, T. Sandev, Random resetting in search problems. arXiv [Preprint] (2023). 10.48550/arXiv.2310.12057 (Accessed 19 October 2023).

[19] S. Redner, A Guide to First-Passage Processes (Cambridge University Press, 2001).

[20] J. J. Hopfield, Kinetic proofreading: A new mechanism for reducing errors in biosynthetic processes requiring high specificity. Proc. Natl. Acad. Sci. U.S.A. **71**, 4135–4139 (1974). 4530290 10.1073/pnas.71.10.4135 PMC434344

[21] M. Luby, A. Sinclair, D. Zuckerman, “Optimal speedup of las vegas algorithms” in *[1993] The 2nd Israel Symposium on Theory and Computing Systems* (IEEE Computer Society Press, Los Alamitos, CA, 1993).

[22] C. P. Gomes, B. Selman, H. Kautz, “Boosting combinatorial search through randomization” in *Proceedings of the Fifteenth National/Tenth Conference on Artificial Intelligence/Innovative Applications of Artificial Intelligence, AAAI ’98/IAAI ’98*, J. Mostow, C. Rich, Eds. (American Association for Artificial Intelligence, USA, 1998), pp. 431–437.

[23] D. Boyer, C. Solis-Salas, Random walks with preferential relocations to places visited in the past and their application to biology. Phys. Rev. Lett. **112**, 240601 (2014). 24996076 10.1103/PhysRevLett.112.240601

[24] G. Mercado-Vásquez, D. Boyer, Lotka-volterra systems with stochastic resetting. J. Phys. A Math. Theor. **51**, 405601 (2018).

[25] A. Pal, L. Kuśmierz, S. Reuveni, Search with home returns provides advantage under high uncertainty. Phys. Rev. Res. **2**, 043174 (2020).

[26] G. Bel, B. Munsky, I. Nemenman, The simplicity of completion time distributions for common complex biochemical processes. Phys. Biol. **7**, 016003 (2009). 20026876 10.1088/1478-3975/7/1/016003

[27] S. Reuveni, M. Urbakh, J. Klafter, Role of substrate unbinding in michaelis-menten enzymatic reactions. Proc. Natl. Acad. Sci. U.S.A. **111**, 4391–4396 (2014). 24616494 10.1073/pnas.1318122111 PMC3970482

[28] O. Blumer, S. Reuveni, B. Hirshberg, Combining stochastic resetting with metadynamics to speed-up molecular dynamics simulations. Nat. Commun. **15**, 240 (2024). 38172126 10.1038/s41467-023-44528-w PMC10764788

[29] B. Mukherjee, K. Sengupta, S. N. Majumdar, Quantum dynamics with stochastic reset. Phys. Rev. B **98**, 104309 (2018).

[30] D. C. Rose, H. Touchette, I. Lesanovsky, J. P. Garrahan, Spectral properties of simple classical and quantum reset processes. Phys. Rev. E **98**, 022129 (2018). 30253565 10.1103/PhysRevE.98.022129

[31] S. Belan, V. Parfenyev, Optimality and universality in quantum zeno dynamics. New J. Phys. **22**, 073065 (2020).

[32] A. Riera-Campeny, J. Ollé, A. Masó-Puigdellosas, Measurement-induced resetting in open quantum systems. arXiv [Preprint] (2020). 10.48550/arXiv.2011.04403 (Accessed 13 January 2021).

[33] G. Perfetto, F. Carollo, M. Magoni, I. Lesanovsky, Designing nonequilibrium states of quantum matter through stochastic resetting. Phys. Rev. B **104**, L180302 (2021).

[34] G. Perfetto, F. Carollo, I. Lesanovsky, Thermodynamics of quantum-jump trajectories of open quantum systems subject to stochastic resetting. SciPost Phys. **13**, 079 (2022).

[35] X. Turkeshi, M. Dalmonte, R. Fazio, M. Schirò, Entanglement transitions from stochastic resetting of non-hermitian quasiparticles. Phys. Rev. B **105**, L241114 (2022).

[36] D. Das, S. Dattagupta, S. Gupta, Quantum unitary evolution interspersed with repeated non-unitary interactions at random times: The method of stochastic liouville equation, and two examples of interactions in the context of a tight-binding chain. J. Stat. Mech. Theory Exp. **2022**, 053101 (2022).

[37] M. Magoni, F. Carollo, G. Perfetto, I. Lesanovsky, Emergent quantum correlations and collective behavior in noninteracting quantum systems subject to stochastic resetting. Phys. Rev. A **106**, 052210 (2022).

[38] R. Yin, E. Barkai, Restart expedites quantum walk hitting times. Phys. Rev. Lett. **130**, 050802 (2023). 36800468 10.1103/PhysRevLett.130.050802

[39] R. Yin, Q. Wang, E. Barkai, Instability in the quantum restart problem. Phys. Rev. E **109**, 064150 (2024). 39020895 10.1103/PhysRevE.109.064150

[40] R. Modak, S. Aravinda, Non-hermitian description of sharp quantum resetting. arXiv [Preprint] (2023). 10.48550/arXiv.2303.03790 (Accessed 20 March 2023).

[41] A. Acharya, S. Gupta, Tight-binding model subject to conditional resets at random times. Phys. Rev. E **108**, 064125 (2023). 38243552 10.1103/PhysRevE.108.064125

[42] M. Kulkarni, S. N. Majumdar, First detection probability in quantum resetting via random projective measurements. J. Phys. A Math. Theor. **56**, 385003 (2023).

[43] M. Kulkarni, S. N. Majumdar, Generating entanglement by quantum resetting. Phys. Rev. A **108**, 062210 (2023).

[44] P. Chatterjee, S. Aravinda, R. Modak, Quest for optimal quantum resetting: Protocols for a particle on a chain. *Phys. Rev*. E **110**, 034132 (2024). 10.1103/PhysRevE.110.034132 39425336

[45] D. A. Puente, F. Motzoi, T. Calarco, G. Morigi, M. Rizzi, Quantum state preparation via engineered ancilla resetting. Quantum **8**, 1299 (2024).

[46] F. Liu, Semi-markov processes in open quantum systems. II. Counting statistics with resetting. Phys. Rev. E **108**, 064101 (2023). 38243423 10.1103/PhysRevE.108.064101

[47] F. A. Grünbaum, L. Velázquez, A. H. Werner, R. F. Werner, Recurrence for discrete time unitary evolutions. Commun. Math. Phys. **320**, 543–569 (2013).

[48] H. Krovi, T. A. Brun, Quantum walks with infinite hitting times. Phys. Rev. A **74**, 042334 (2006).

[49] S. Dhar, S. Dasgupta, A. Dhar, Quantum time of arrival distribution in a simple lattice model. J. Phys. A Math. Theor. **48**, 115304 (2015).

[50] S. Dhar, S. Dasgupta, A. Dhar, D. Sen, Detection of a quantum particle on a lattice under repeated projective measurements. Phys. Rev. A **91**, 062115 (2015).

[51] H. Friedman, D. A. Kessler, E. Barkai, Quantum walks: The first detected passage time problem. Phys. Rev. E **95**, 032141 (2017). 28415197 10.1103/PhysRevE.95.032141

[52] F. Thiel, E. Barkai, D. A. Kessler, First detected arrival of a quantum walker on an infinite line. Phys. Rev. Lett. **120**, 040502 (2018). 29437409 10.1103/PhysRevLett.120.040502

[53] T. Nitsche , Probing measurement-induced effects in quantum walks via recurrence. Sci. Adv. **4**, eaar6444 (2018). 29963626 10.1126/sciadv.aar6444 PMC6025909

[54] S. Lahiri, A. Dhar, Return to the origin problem for a particle on a one-dimensional lattice with quasi-zeno dynamics. Phys. Rev. A **99**, 012101 (2019).

[55] R. Yin, K. Ziegler, F. Thiel, E. Barkai, Large fluctuations of the first detected quantum return time. Phys. Rev. Res. **1**, 033086 (2019).

[56] F. Thiel, I. Mualem, D. Meidan, E. Barkai, D. A. Kessler, Dark states of quantum search cause imperfect detection. Phys. Rev. Res. **2**, 043107 (2020).

[57] F. Thiel, I. Mualem, D. A. Kessler, E. Barkai, Uncertainty and symmetry bounds for the quantum total detection probability. Phys. Rev. Res. **2**, 023392 (2020).

[58] P. Kuklinski, Conditional probability distributions of finite absorbing quantum walks. Phys. Rev. A **101**, 032309 (2020).

[59] F. Thiel, I. Mualem, D. Kessler, E. Barkai, Uncertainty relation between detection probability and energy fluctuations. Entropy **23**, 595 (2021). 34064881 10.3390/e23050595 PMC8151696

[60] D. A. Kessler, E. Barkai, K. Ziegler, First-detection time of a quantum state under random probing. Phys. Rev. A **103**, 022222 (2021).

[61] K. Ziegler, E. Barkai, D. A. Kessler, Randomly repeated measurements on quantum systems: Correlations and topological invariants of the quantum evolution. J. Phys. A Math. Theor. **54**, 395302 (2021).

[62] V. Dubey, C. Bernardin, A. Dhar, Quantum dynamics under continuous projective measurements: Non-hermitian description and the continuum-space limit. Phys. Rev. A **103**, 032221 (2021).

[63] A. Didi, E. Barkai, Measurement-induced quantum walks. Phys. Rev. E **105**, 054108 (2022). 35706264 10.1103/PhysRevE.105.054108

[64] Q. Liu, K. Ziegler, D. A. Kessler, E. Barkai, Driving quantum systems with periodic conditional measurements. Phys. Rev. Res. **4**, 023129 (2022).

[65] D. Das, S. Gupta, Quantum random walk and tight-binding model subject to projective measurements at random times. J. Stat. Mech Theory Exp. **2022**, 033212 (2022).

[66] Y. J. Wang, R. Y. Yin, L. Y. Dou, A. N. Zhang, X. B. Song, Quantum first detection of a quantum walker on a perturbed ring. Phys. Rev. Res. **5**, 013202 (2023).

[67] Z. Ni, Y. Zheng, First detection and tunneling time of a quantum walk. Entropy **25**, 1231 (2023). 37628261 10.3390/e25081231 PMC10453060

[68] B. Walter, G. Perfetto, A. Gambassi, Thermodynamic phases in first detected return times of quantum many-body systems. arXiv [Preprint] (2023). 10.48550/arXiv.2311.05585 (Accessed 14 March 2024).

[69] S. Tornow, K. Ziegler, Measurement-induced quantum walks on an IBM quantum computer. Phys. Rev. Res. **5**, 033089 (2023).

[70] L. Laneve, F. Tacchino, I. Tavernelli, On hitting times for general quantum markov processes. Quantum **7**, 1056 (2023).

[71] B. Misra, E. C. G. Sudarshan, The zeno’s paradox in quantum theory. J. Math. Phys. **18**, 756–763 (1977).

[72] M. N. Jayakody, I. L. Paiva, A. Nanayakkara, E. Cohen, Induced on-demand revival in coined quantum walks on infinite *d*-dimensional lattices. Phys. Rev. A **105**, 032413 (2022).

[73] T. R. Gingrich, J. M. Horowitz, Fundamental bounds on first passage time fluctuations for currents. Phys. Rev. Lett. **119**, 170601 (2017). 29219443 10.1103/PhysRevLett.119.170601

[74] J. P. Garrahan, Simple bounds on fluctuations and uncertainty relations for first-passage times of counting observables. Phys. Rev. E **95**, 032134 (2017). 28415371 10.1103/PhysRevE.95.032134

[75] G. Falasco, M. Esposito, Dissipation-time uncertainty relation. Phys. Rev. Lett. **125**, 120604 (2020). 33016734 10.1103/PhysRevLett.125.120604

[76] A. Pal, S. Reuveni, S. Rahav, Thermodynamic uncertainty relation for first-passage times on markov chains. Phys. Rev. Res. **3**, L032034 (2021).

[77] Y. Hasegawa, Thermodynamic uncertainty relation for quantum first-passage processes. Phys. Rev. E **105**, 044127 (2022). 35590682 10.1103/PhysRevE.105.044127

[78] R. Bebon, A. Godec, Controlling uncertainty of empirical first-passage times in the small-sample regime. Phys. Rev. Lett. **131**, 237101 (2023). 38134782 10.1103/PhysRevLett.131.237101

[79] O. L. Bonomo, A. Pal, First passage under restart for discrete space and time: Application to one-dimensional confined lattice random walks. Phys. Rev. E **103**, 052129 (2021). 34134266 10.1103/PhysRevE.103.052129

[80] IBM Noise Models (2024). https://docs.quantum.ibm.com/guides/build-noise-models. Accessed 8 August 2024.

[81] M. Kac, On distributions of certain wiener functionals. Trans. Am. Math. Soc. **65**, 1–13 (1949).

[82] C. Cohen-Tannoudji, B. Diu, F. Laloë, Quantum Mechanics, Volume 1: Basic Concepts, Tools, and Applications (Wiley, 2019).

[83] S. E. Harris, Electromagnetically induced transparency. Phys. Today **50**, 36–42 (1997).

[84] C. Liu , Observation of coherent optical information storage in an atomic medium using halted light pulses. Nature **409**, 490–493 (2001). 11206540 10.1038/35054017

[85] M. Fleischhauer, A. Imamoglu, J. P. Marangos, Electromagnetically induced transparency: Optics in coherent media. Rev. Mod. Phys. **77**, 633–673 (2005).

[86] G. Alzetta , An experimental method for the observation of r.f. transitions and laser beat resonances in oriented Na vapour. Nuovo Cimento **36**, 5–20 (1976).

[87] R. M. Whitley, C. R. Stroud, Double optical resonance. Phys. Rev. A **14**, 1498–1513 (1976).

[88] E. Arimondo, G. Orriols, Nonabsorbing atomic coherences by coherent two-photon transitions in a three-level optical pumping. Lett. Nuovo Cimento **17**, 333–338 (1976).

[89] H. R. Gray, R. M. Whitley, C. R. Stroud, Coherent trapping of atomic populations. Opt. Lett. **3**, 218–220 (1978). 19684752 10.1364/ol.3.000218

[90] B. Lounis, C. Cohen-Tannoudji, Coherent population trapping and fano profiles. J. Phys. II Fr. **2**, 579–592 (1992).

[91] E. Arimondo, *V Coherent Population Trapping in Laser Spectroscopy Progress in Optics*, E. Wolf, Ed. (Elsevier, 1996), vol. 35, pp. 257–354.

[92] A. Aspect, E. Arimondo, R. Kaiser, N. Vansteenkiste, C. Cohen-Tannoudji, Laser cooling below the one-photon recoil energy by velocity-selective coherent population trapping. Phys. Rev. Lett. **61**, 826–829 (1988). 10039440 10.1103/PhysRevLett.61.826

[93] J. Lawall , Three-dimensional laser cooling of helium beyond the single-photon recoil limit. Phys. Rev. Lett. **75**, 4194–4197 (1995). 10059843 10.1103/PhysRevLett.75.4194

[94] D. R. Fernandes , Sub-doppler laser cooling of fermionic 40k atoms in three-dimensional gray optical molasses. Europhys. Lett. **100**, 63001 (2012).

[95] E. Bäumer *et al*., Efficient long-range entanglement using dynamic circuits. *PRX Quantum* **5**, 030339 (2024).

[96] S. Krinner , Realizing repeated quantum error correction in a distance-three surface code. Nature **605**, 669–674 (2022). 35614249 10.1038/s41586-022-04566-8

[97] B. Skinner, J. Ruhman, A. Nahum, Measurement-induced phase transitions in the dynamics of entanglement. Phys. Rev. X **9**, 031009 (2019).

[98] J. M. Koh , Measurement-induced entanglement phase transition on a superconducting quantum processor with mid-circuit readout. Nat. Phys. **19**, 1314–1319 (2023).

[99] B. J. J. Khor, M. Wampler, G. Refael, I. Klich, Measurement-induced chirality: Diffusion and disorder. Phys. Rev. B **108**, 214305 (2023).

[100] F. Schmolke, E. Lutz, Measurement-induced quantum synchronization and multiplexing. Phys. Rev. Lett. **132**, 010402 (2024). 38242665 10.1103/PhysRevLett.132.010402

[101] A. Montanaro, Quantum-walk speedup of backtracking algorithms. Theory Comput. **14**, 1–24 (2018).

[102] R. Yin, Q. Wang, S. Tornow, E. Barkai, Data from “Restart uncertainty relation for monitored quantum dynamics.” Zenodo. 10.5281/zenodo.13327746. Deposited 5 January 2024. PMC11725946 39746039

