# Supplementary material for: Restart uncertainty relation for monitored quantum dynamics
Source: Proc Natl Acad Sci U S A. 2025 Jan 2;122(1):e2402912121. doi: 10.1073/pnas.2402912121 (PMC11725946; doi:10.1073/pnas.2402912121)
Supplement: Supplementary file 1 — Appendix 01 (PDF) [file pnas.2402912121.sapp.pdf]

Supporting Information for

Restart uncertainty relation for monitored quantum dynamics

Ruoyu Yin, Qingyuan Wang, Sabine Tornow, Eli Barkai

Ruoyu Yin, Qingyuan Wang, Sabine Tornow, Eli Barkai.
E-mail: yinruoy@biu.ac.il; qingwqy@gmail.com; sabine.tornow@unibw.de; Eli.Barkai@biu.ac.il

This PDF file includes:

Supporting text
Figs. S1 to S19
Tables S1 to S2
SI References

Supporting Information Text

1. Experimental mean hitting time under restart and noise model simulations

We now address the origin of the vertical shift observed in the experimentally derived restarted mean hitting time, $\langle n_R \rangle$, as presented in Fig. 3(b) in the main text. Given the observed strong concordance between experimental results and exact calculations of $\langle n \rangle_{\text{Con}}$, as shown in Fig. 3(a), we postulate that the vertical shift primarily stems from the second term in equation (4), $T_R[1 - P_{\text{det}}(T_R)]/P_{\text{det}}(T_R)$, in the context of quantum hitting time with restarts.

To substantiate this hypothesis, we illustrate the detection probability $P_{\text{det}}(T_R)$ with $T_R = 20$ in Fig. S1, obtained from experiments, exact calculation (using equation (2)), the theory (see below) and simulations (using IBM quantum simulators). Using equation (8) in the main text, and $a(\zeta_{\max}) = (1 - |\zeta_{\max}|^2)^2$, we get the theory

$$P_{\text{det}}(T_R) = \sum_{n=1}^{T_R} F_n \simeq 1 - (1 - |\zeta_{\max}|^2) e^{-T_R(1-|\zeta_{\max}|^2)}, \quad [1]$$

The figure reveals a small discrepancy between the experimental and exact/theoretical/simulated results, suggesting the presence of measurement noise. More specifically, consider τ far from resonance at $\tau = 2\pi/3$, the theory predicts $P_{\text{det}} \to 1$, namely within 20 measurements the click yes is nearly surely guaranteed. The result from the experiment is $P_{\text{det}} \simeq 0.99$, namely the deviation from theory is merely one percent. However, using $P_{\text{det}} = 0.99$ we get for $T_R = 20$, $T_R(1 - P_{\text{det}})/P_{\text{det}} \simeq 0.2$, while the theory predicts a nearly zero value. This means that $\langle n_R \rangle$ is expected to be shifted by roughly 0.2 due to the small error in P_{det}. The issue here is that a small variation in P_{det}, or the order of one percent, can lead to a small shift for the mean return time, since the second term in equation (4) is linear in T_R. The larger T_R is the bigger we expect the shift in $\langle n_R \rangle$ to be. Remarkably, the shift of P_{det} is roughly one percent for all τ, see Fig. S1. It follows that the shift of $\langle n_R \rangle$ due to the small errors in P_{det}, is roughly 0.2. To test this we plot in Fig. S2, $\langle n_R \rangle -$ shift, where as mentioned for $T_R = 20$, the expected shift is 0.2. Now the theory and exact results reach an excellent agreement with the experimental results.

It is crucial to highlight that in the data analysis of the conditional mean $\langle n \rangle_{\text{Con}}$, as described by equation (3), the noise in $P_{\text{det}}(T_R)$ is effectively mitigated or eliminated through the exclusion of non-detection trajectories, as $P_{\text{det}}(T_R) = \sum_{n=1}^{T_R} F_n$ appears in the denominator of the equation This further explains the observed perfect alignment between the theoretical prediction and experimental results for $\langle n \rangle_{\text{Con}}$.

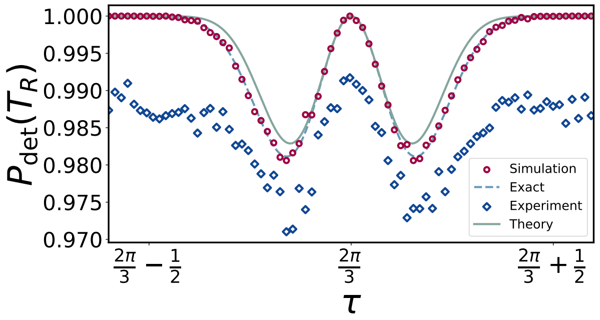

Fig. S1. The sample $P_{\text{det}}(T_R) = \sum_{n=1}^{T_R} F_n$, for $T_R = 20$, for quantum hitting times under restarts versus τ is estimated from the experimental data. The red circles are obtained from IBM quantum simulators, and the blue dots are from experiments on the IBM quantum computer. The green solid/dashed line represents the theory equation (1)/exact results (using equation (2)). We see that experimental results are shifted compared to theory, revealing roughly one percent error in the measurement. This error gives rise to the shift observed in Fig. S2. The model here is a tight-binding three-site ring as in Fig. S2.

We now analyze the cause of errors in P_{det}, which is related to quantum error and noise and its consequent leakage.

Leakage. In the implementation of the three-site ring model using mid-circuit measurements on the IBM quantum computer, we employed a two-qubit system. As mentioned in the main text, in our model, the states of the triangle model, $|0\rangle$, $|1\rangle$ and $|2\rangle$, are mapped to the qubit states $|01\rangle$, $|10\rangle$ and $|00\rangle$, respectively. Theoretically, the state $|11\rangle$ is decoupled from the other states. However, practical experiments on a quantum computer demonstrated leakage from the utilized qubit states $(|00\rangle, |01\rangle, |10\rangle)$ to the excluded state $(|11\rangle)$, as mentioned in Materials and Methods. Note that after twenty measurements (which is the length of our experiment), we find leakage of one percent, hence while clearly an important issue, the leakage is not large. We anticipate an increase of leakage as we increase T_R and possibly also if the size of the system grows as more noise will be present. This in turn will affect the mean recurrence time. We want to note that the leakage in our problem is merely one of the consequences of noise existing on current quantum processors.

Noise on IBM quantum processors. Noise in current quantum computing platforms is a critical challenge impacting computational accuracy and reliability. Quantum noise arises from various sources, including environmental decoherence, control errors, and imperfect quantum gate operations. As mentioned, the leakage in our problem is merely one of the consequences of noise existing on current quantum processors. Fortunately, the IBM quantum computing platform provides various noise models, with which one may predict behaviors of simulated quantum dynamics, on noisy quantum circuits (1).

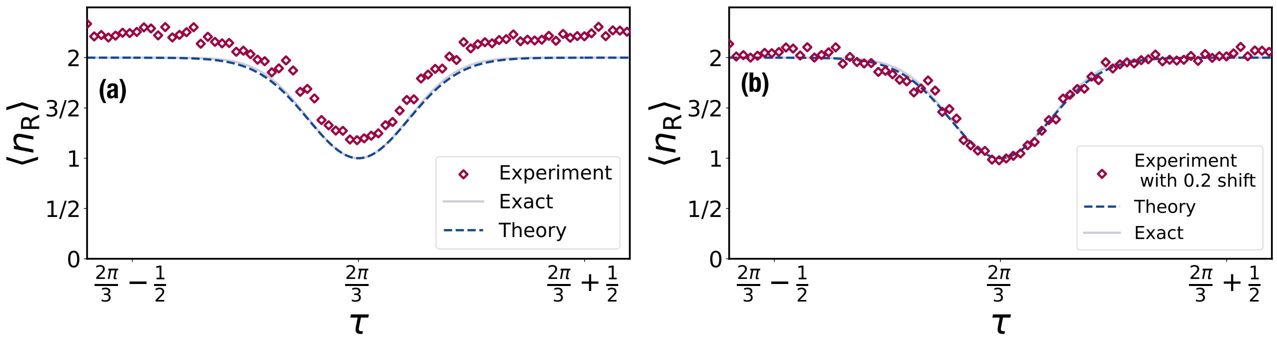

Fig. S2. (a) The mean hitting time, in units of τ, under restart, $\langle n_R \rangle$, as a function of τ. We compare the exact results (obtained by equation (4), gray solid line), the theory (obtained by equation (7), blue dashed line) and experimental results on a quantum computer (squares) for $T_R = 20$. We observe the vertical shift between the experimental and exact results, which is due to noise in the quantum computer. The model here, as in Figs. 2,3 in the main text, is a tight-binding three-site ring (equation (15) with $L = 3$). (b) The down-shifted experimental $\langle n_R \rangle$ (squares) compared with the theory (the dashed line obtained from equation (7)) and exact results (the solid line obtained with equation (4)). With the experimental data shifting downward by 0.20 explained by Fig. S1, the theory agrees nicely with the experimental results.

Utilizing two common noise models, i.e. bit-flip error, and thermal relaxation (1), we observed clear effects of noise on the mean recurrence time for the monitored quantum dynamics. As seen in Figure S3, an upward shift of the theoretical $\langle n_R \rangle$ is induced by these noise models, yet the resonance dip remains visible. Both noise accumulates with measurements and evolution time, resulting in more pronounced effects as T_R increases, i.e. compare shifts on the left and right panels in Figure S3. It is noteworthy that our simulation is based on the same quantum circuit which is employed to conduct the IBM experiment in the main text, namely we have two qubits and hence four states that evolve on the noisy circuit. Therefore, we believe that our simulation is a proper estimator for a noisy quantum computing platform.

Now we present details of the noise models, including their physical implication, parameters and additional numerical results. As mentioned above, we chose two noise models: the bit-flip error, and the thermal relaxation (1).

The bit-flip error noise model represents a quantum error that probabilistically flips a qubit state, i.e. from $|0\rangle$ to $|1\rangle$ or vice versa, serving as a fundamental noise channel that explains state transitions. This error might be led by gate imperfections, interactions with nearby qubits, etc. The bit-flip error noise model is characterized by the following parameters and we extract from (1) the description:

- For a single-qubit gate, invert the qubit's state with a probability of p_{gate1}.
- For a two-qubit gate, introduce single-qubit errors independently to each qubit.
- When resetting a qubit, set it to 1 instead of 0 with a probability of p_{reset}.
- During a qubit measurement, flip the qubit's state with a probability of p_{meas}.

We note that this model captures errors caused by measurements, as indicated by the parameter p_{meas}, which is in line with our setup of repeated measurements.

The thermal relaxation noise model describes how a qubit state naturally decays over time due to interactions with its environment. This model encompasses two primary processes, energy relaxation or amplitude damping (also called T_1 relaxation), and dephasing (or T_2 relaxation). The physical meaning of the parameters T_1, T_2 is the following:

- T_1 relaxation is the process by which a qubit in the excited state $|1\rangle$ decays to the ground state $|0\rangle$. This represents the loss of energy from the qubit to the environment. Over time, the probability of the qubit being in $|1\rangle$ decreases, leading to a loss of coherence in quantum computations.
- T_2 relaxation process describes the loss of phase information without a change in the energy level of the qubit, e.g. the relative phase between $|0\rangle$ and $|1\rangle$ may change unpredictably, leading to decoherence.
- Longer T_1, T_2 times imply that the qubits can maintain their quantum state for longer periods, namely higher fidelity.

See the implementation of the two noise models using *Qiskit* in (1).

For each noise model, we choose three set of parameter values, denoted as "strong","moderate" and"weak" according to the noise strength, as specified in Table S1. With these choices of parameters, we present in Figures S4,S5 the corresponding behaviors of the mean recurrence time. As expected, intensifying noise leads to more pronounced results, e.g. larger upward shift and increasingly diminishing resonance. For the bit-flip error, both shift and diminishing resonance are witnessed, but the resonance dip remains visible, while for the thermal relaxation noise, we mainly find the upward shift.

Table S1. Parameters for the noise models used in Figures S4,S5.

Noise models	Bit-flip error	Thermal relaxation
"Strong"	$p_{\text{gate1}} = 0.005$, $p_{\text{reset}} = 0.003$, $p_{\text{meas}} = 0.01$	T_1(microsec) $\in [100, 20]$, T_2(microsec) $\in [140, 20]$
"Moderate"	$p_{\text{gate1}} = 0.001$, $p_{\text{reset}} = 0.001$, $p_{\text{meas}} = 0.01$	T_1(microsec) $\in [250, 50]$, T_2(microsec) $\in [350, 50]$
"Weak"	$p_{\text{gate1}} = 0.0005$, $p_{\text{reset}} = 0.0005$, $p_{\text{meas}} = 0.001$	T_1(microsec) $\in [500, 100]$, T_2(microsec) $\in [700, 100]$

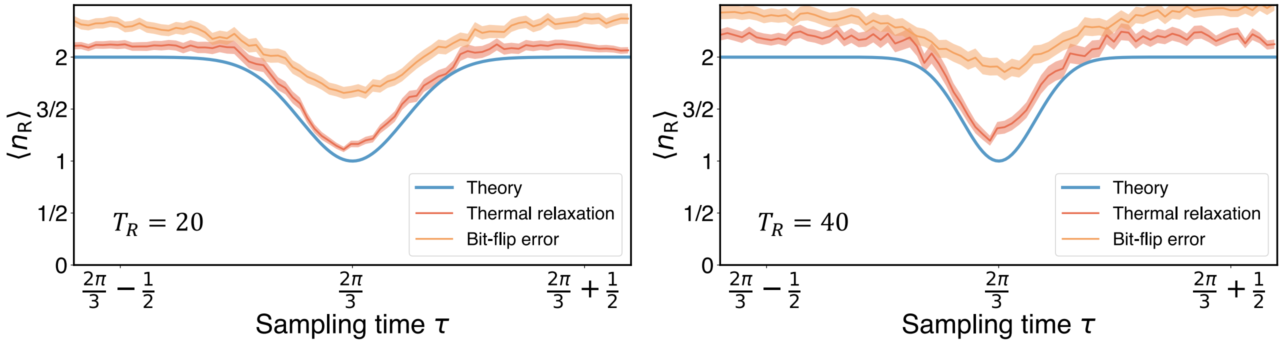

Fig. S3. The effects of noise on the mean recurrence time for the three-site ring model. We chose two common noise models provided by the IBM quantum computing platform, namely the bit-flip error and thermal relaxation noise models (1), with parameter values chosen to align with the IBM technical document (see Ref. (1) for technical details). The blue curve represents the theoretical $\langle n_R \rangle$ with no noise. A vertical shift is witnessed for the thermal relaxation noise, while an additional diminishing resonance is presented for the bit-flip error. These noise-induced effects are more pronounced for a longer restart time, since the noise accumulates with measurement time. The results are obtained using IBM simulators. For bit-flip error, we choose "strong", and for thermal relaxation, we choose "moderate" noise levels (see parameters in Table S1).

2. Rigorous proof of uncertainty principle

We will provide a rigorous proof for the above uncertainty relations, equations (6,7) in the main text. To do so we will find F_n in the large n limit. We also find an exact expression for F_n. In the following derivation, we note that equations (2-12) are not new. The expression inside the bracket in equation (2) can be rewritten as (2),

$$\phi_n = \langle 0| \hat{U}(n\tau) |0\rangle - \sum_{m=1}^{n-1} \langle 0| \hat{U}((n-m)\tau) |0\rangle \, \phi_m. \quad [2]$$

Here ϕ_n is the first detection amplitude, and $F_n = |\phi_n|^2$. equation (2) is also called the quantum renewal equation $|0\rangle$ is the initial and also the target state of the quantum walker. In our examples, the target state is a node on the graph, and since we have in these examples translational invariance, any node will hold. Since equation (2) has a convolution term, applying the Z transform, namely,

$$\tilde{\phi}(z) := \sum_{z=1}^{\infty} z^n \phi_n, \quad [3]$$

we obtain the generating function (2)

$$\tilde{\phi}(z) = \frac{\langle 0| \hat{\mathcal{U}}(z) |0\rangle}{1 + \langle 0| \hat{\mathcal{U}}(z) |0\rangle}, \quad [4]$$

where $\hat{\mathcal{U}}(z) := \sum_{n=1}^{\infty} z^n \hat{U}(n\tau) = ze^{-iH\tau}/(1 - ze^{-iH\tau})$. The generating function is a useful tool with which we may obtain many results, the inversion formula

$$\phi_n = \frac{1}{2\pi i} \oint_{|z|=1} \frac{dz}{z^{n+1}} \tilde{\phi}(z) \quad [5]$$

provides a formal solution to the problem. Via spectral decomposition of equation (4) (into the energy eigenbasis), we have (2)

$$\tilde{\phi}(z) = \frac{\sum_{k=1}^{w} \sum_{l=1}^{g_k} |\langle 0|E_{kl}\rangle|^2 ze^{-iE_k\tau}/(1 - ze^{-iE_k\tau})}{\sum_{k=1}^{w} \sum_{l=1}^{g_k} |\langle 0|E_{kl}\rangle|^2 (1 - ze^{-iE_k\tau})^{-1}}, \quad [6]$$

where w is the number of distinct energy phase factors $\exp(-iE_k\tau)$ with non-zero overlap $\sum_{l=1}^{g_k} |\langle 0|E_{kl}\rangle|^2$, g_k is the degeneracy of E_k ($g_k \geq 2$ means degenerate energy levels), and $|E_{kl}\rangle$ are the eigenstates corresponding to E_k. equation (6) can be rewritten

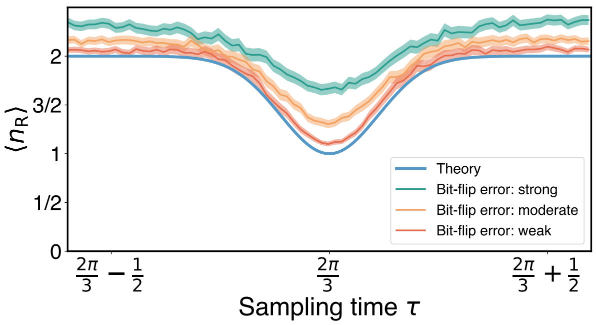

Fig. S4. The mean recurrence time exhibits diminishing resonance when the circuit implementation is incorporated with bit-flip errors. Here the restart time $T_R = 20$. We see that stronger noise leads to more pronounced effects, but the resonance, as well as the constant mean recurrence time far from the resonance, are not ruined by noise. We also see a shift upwards, compared to theory, as explained in the text. See Table S1 for parameters corresponding to "strong", "moderate" and "weak" noise.

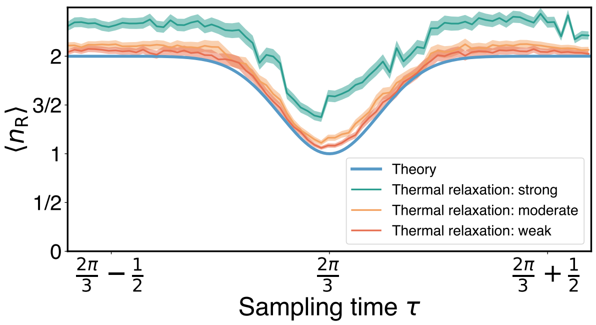

Fig. S5. The mean recurrence time is shifted vertically when the circuit implementation is incorporated with the thermal relaxation model. Here the restart time $T_R = 20$. The resonance, as well as the constant $\langle n_R \rangle$ far from the resonance, are relatively robust to this type of noise, except for an upward shift increasing with stronger noise. See Table S1 for parameters corresponding to "strong", "moderate" and "weak" noise.

as

$$\tilde{\phi}(z) = \frac{\mathcal{N}(z)}{\mathcal{D}(z)},$$
$$\text{with } \mathcal{N}(z) = z \sum_{k=1}^{w} \sum_{l}^{g_k} |\langle 0|E_{kl}\rangle|^2 \prod_{j=1, j\neq k}^{w} \left(z - e^{iE_j\tau}\right), \qquad [7]$$
$$\mathcal{D}(z) = \sum_{k=1}^{w} e^{iE_k\tau} \sum_{l}^{g_k} |\langle 0|E_{kl}\rangle|^2 \prod_{j=1, j\neq k}^{w} \left(z - e^{iE_j\tau}\right).$$

And one can prove the relation (2)

$$\mathcal{D}(z) = (-1)^{w-1} e^{i\chi} z^w \left[\mathcal{N}\left(1/z^*\right)\right]^*, \qquad [8]$$

where $\chi = \sum_{k=1}^{w} \tau E_k$, and the superscript "$*$" means complex conjugate. Then we can factorize $\tilde{\phi}(z)$ as (3)

$$\tilde{\phi}(z) = z e^{-i\chi} \prod_{i=1}^{w-1} \frac{z - z_i}{z_i^*(z - 1/z_i^*)}, \qquad [9]$$

where $\{z_i\}$ are the zeros of $\mathcal{N}(z)$ or $\tilde{\phi}(z)$. These zeros are located inside the unit circle in the complex plane. As mentioned, they are also the conjugate of the eigenvalues of the survival operator $\mathcal{S} = \left(1 - \hat{D}\right)\hat{U}(\tau)$. This can be proven by applying the matrix determinant lemma to the characteristic polynomial of $\mathcal{S}$ (4), namely,

$$0 = \det\left[\zeta\mathbb{1} - \mathcal{S}\right] = \det[\zeta\mathbb{1} - \hat{U}(\tau) + |0\rangle\langle 0|\,\hat{U}(\tau)] = \det[\zeta\mathbb{1} - \hat{U}(\tau)]\,\langle 0|[\zeta\mathbb{1} - \hat{U}(\tau)]^{-1}|0\rangle\,. \qquad [10]$$

The term $\langle 0|[\zeta\mathbb{1} - \hat{U}(\tau)]^{-1}|0\rangle$ can be spectrally decomposed as $\sum_{k=1}^{w} \sum_{l=1}^{g_k} |\langle 0|E_{kl}\rangle|^2 \left[1/(\zeta - e^{-iE_k\tau})\right]$, which, equal to 0, gives the eigenvalues of $\mathcal{S}$, $\{\zeta_i\}$, inside the unit disk, that are conjugate of the zeros of $\tilde{\phi}(z)$ (excluding the trivial zero $z = 0$). Namely,

$$\zeta_i = z_i^*. \qquad [11]$$

We note here that the mean hitting time $\langle n\rangle$ (for infinite measurements, i.e. $T_R=\infty$) can be computed by

$$\langle n\rangle = \frac{1}{2\pi i}\oint_{|z|=1} \partial_z \ln\left[\tilde{\phi}(z)\right]\,\mathrm{d}z, \tag{12}$$

which directly gives $\langle n\rangle = w$ using equation (9). Namely, the mean $\langle n\rangle$ is identical to the number of zeros of $\tilde{\phi}(z)$, *inside the unit disk*.

Substituting equation (9) into equation (5) and using the residue theorem yield

$$\phi_n = e^{-i\chi}\sum_{j=1}^{w-1}\left(z_j^*\right)^{n-1}\left(\frac{1}{z_j^*}-z_j\right)\prod_{k\neq j}\frac{z_j^*(1/z_j^*-z_k)}{z_k^*-z_j^*}. \tag{13}$$

Let $z_j=\rho_j\exp(i\theta_j)$, i.e. $\rho_j=|z_j|=|\zeta_i|$, $\theta_j=\arg(z_j)\in[0,2\pi)$, and further simplification gives

$$F_n = |\phi_n|^2 = \sum_{j,k=1}^{w-1}\frac{\alpha_j\alpha_k^*}{\beta_j\beta_k^*}\left(\rho_j\rho_k\right)^n e^{in\Theta_{jk}}, \tag{14}$$

where $\Theta_{jk}=\theta_k-\theta_j\in[0,2\pi)$, and

$$\frac{\alpha_j}{\beta_j} = \frac{\prod_i\left(1/z_i^*\right)\left(1/z_j^*-z_i\right)}{\prod_{i\neq j}\left(1/z_j^*-1/z_i^*\right)}. \tag{15}$$

Hence equation (14) has $(w-1)^2$ terms. Due to the invariance under the switching between j and k in equation (14), the fact that F_n is real is guaranteed by the appearance of paired conjugate terms.

$$F_n \sim a_{\max}\rho_{\max}^{2n} = a_{\max}\left|z_{\max}\right|^{2n} = a_{\max}\left|\zeta_{\max}\right|^{2n}, \tag{16}$$

where $a_{\max}=|\alpha_{\max}/\beta_{\max}|^2$. Using equation (15), and $\rho_{\max}=|\zeta_{\max}|=1-\varepsilon\to 1$, we have

$$a_{\max} = \left|\frac{\alpha_{\max}}{\beta_{\max}}\right|^2 = \left|\frac{1}{z_{\max}^*}\left(\frac{1}{z_{\max}^*}-z_{\max}\right)\right|^2\prod_i{}'\left|\frac{1/z_{\max}^*-z_i}{(z_i^*)\left(1/z_{\max}^*-1/z_i^*\right)}\right|^2 \sim \left(1-|z_{\max}|^{-2}\right)^2\prod_i{}'\left|\frac{1-z_i}{z_i^*-1}\right|^2 \sim \left(1-\rho_{\max}^2\right)^2, \tag{17}$$

where $\prod_i'$ means multiplication over all ii except for $z_i=z_{\max}$. Therefore, we get a universal formula for F_n's tail, in the vicinity of the transition or phase factors matching, namely,

$$F_n \sim \left(1-\rho_{\max}^2\right)^2\rho_{\max}^{2n} = \left(1-|\zeta_{\max}|^2\right)^2|\zeta_{\max}|^{2n}, \tag{18}$$

which confirms rigorously the validity of equation (8). We have assumed that a gap exists between the maximum $|\zeta_{\max}|$ and other zeros of $\mathcal{N}(z)$ in the system. Note: All along we assumed that the Hilbert space is finite, otherwise the spectrum becomes degenerate. Finally, with equation (18) we derive our main results in equations (6,7). We want to note again that $\zeta_{\max}$ is unique in our work.

3. Dependence of restart uncertainty relation on system size

We now discuss the relation between the restart uncertainty principle and the size of the system. Recall that we use the notation $H\,|E_{k,l}\rangle = E_k\,|E_{k,l}\rangle$ where H is the Hamiltonian, l is an index that accounts for possible degeneracy of the energy level. Then when two energy phases match $\exp(-iE_-\tau)\sim\exp(-iE_+\tau)$ for a pair of energies E_+ and E_-, where τ is the sampling time, we find a resonance in the mean number of measurement till the first detection. In particular, using the equations (13) and (14) in the main text, we state the uncertainty related to system energy,

$$\langle n\rangle_{\text{Con}} = w-\left[1+\lambda T_R(\widetilde{\Delta E\tau})^2\right]\exp\left[-\lambda T_R(\widetilde{\Delta E\tau})^2\right], \tag{19}$$

$$\langle n_R\rangle = w-\exp\left[-\lambda T_R(\widetilde{\Delta E\tau})^2\right], \tag{20}$$

where $\langle n\rangle_{\text{Con}}$ is the conditional mean, $\langle n_R\rangle$ is the restarted mean and w is the topological number which is determined by the distinct energy eigenvalues of the system. Later we will only focus on the restarted mean since similar behaviors are found for the conditional mean. The parameters $\lambda=p_+p_-/(p_++p_-)^3$ with the overlaps $p_\pm=\sum_l^{g_\pm}|\langle 0|E_{\pm,l}\rangle|^2$ ($g_\pm$ is the degeneracy of the energy level $E_\pm$, and the location of the target $x_{\mathrm{d}}=0$ as in the main text), and

$$\widetilde{\Delta E\tau} := \tau|E_+-E_-| \mod 2\pi. \tag{21}$$

Hence, we need to find out how the size of a system will affect its energy levels and energy eigenstates (which determine the overlaps $p_\pm$ and λ). Since energies depend on system size, so will the resonances, however, additionally λ is also generally size-dependent. This implies rich types of physical behaviors as system size is changed.

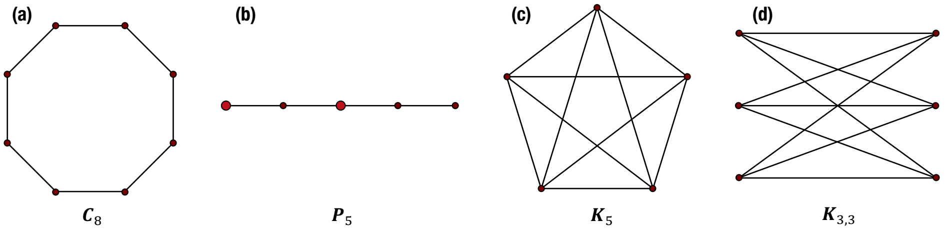

Fig. S6. Schematics for the graphs under investigation. From the left to the right are the examples: (a) ring of size L, C_L, in the figure $L = 8$, (b) finite segment of size L, P_L, in the figure $L = 5$, (c) complete graph of size L, K_L, in the figure $L = 5$, (d) complete bipartite graph $K_{m,n}$, in the figure $m = n = 3$, and the size is $L = m + n = 6$. We will verify our theory using different sizes. For graph (b), the target site will be chosen at the end or the middle (marked with larger vertices), which leads to non-identical resonance widths.

Table S2. The winding number w, the maximal difference between energies $\Delta E_{\mathrm{m}} = E_{\max} - E_{\min}$, used in $\widetilde{\Delta E\tau} = (\tau\Delta E_{\mathrm{m}} \mod 2\pi)$, and the parameter $\lambda = p_+p_-/(p_+ + p_-)^3$, for different graphs with L vertices, including even rings C_L, complete graphs K_L, finite segments P_L, and complete bipartite graphs $K_{L/2,L/2}$. Only for the segment, i.e. the P_L graph, the location of the target, denoted x_{d}, is important.

Graph	$\boldsymbol{C}_L$, L is even	$\boldsymbol{K}_L$	$\boldsymbol{P}_L$, L is odd	$\boldsymbol{K}_{\frac{L}{2},\frac{L}{2}}$
$\boldsymbol{w}$	$L/2+1$	2	L, $x_{\mathrm{d}} = 1$;<br>$(L+1)/2$, $x_{\mathrm{d}} = (L+1)/2$	3
$\boldsymbol{\Delta}E_{\mathrm{m}}$	4γ	1	$4\gamma\cos[\pi/(L+1)]$	γL
$\boldsymbol{\lambda}$	$L/8$	$(L-1)/L^2$	$(L+1)^3/16\pi^2$, $x_{\mathrm{d}} = 1$;<br>$(L+1)/16$, $x_{\mathrm{d}} = (L+1)/2$	$L/8$

Without delving into details, we have summarized in Table S2, the values of parameters in equations (19) and (20), for different graphs, with the resonance chosen at $\exp(-iE_{\max}\tau) \sim \exp(-iE_{\min}\tau)$, where $E_{\max}$ and $E_{\min}$ are the maximum and minimum of energies of the system respectively. It is clearly shown that different graph structures lead to various relations between the width of transitions and the system size L.

Ring models. We start with the ring model (Figure S6(a)), which is used for demonstration purposes in the manuscript. Energies of the ring model of size L are $E_k = -2\gamma\cos\theta_k$ with $\theta_k = 2\pi k/L$ and $k = 0, 1, 2, \ldots, L-1$ (see equation (17) in the text), and overlaps are $|\langle x|E_k\rangle|^2 = 1/L$ for any node x , the broadening can be easily associated with the system size L (assuming even L). See Figure S7(a-b) for a schematics of its energy structures, where the parity of L plays a role. We start the discussion where the pair of energies is $E_{\max}$ and $E_{\min}$, and then consider the case when we chose the energy difference between the the ground state and the first excited state (this is based on odd ring, otherwise the transition will be $w \to w-2$ which is left for future study).

For the resonance between the ground state and the highest energy state, where phase factors $\{e^{-i2\gamma\tau}, e^{i2\gamma\tau}\}$ merge, we have $\widetilde{\Delta E\tau} = \tau\Delta E_{\mathrm{m}} \mod 2\pi = 4\gamma\tau \mod 2\pi$ (and now we set γ as 1), and $\Delta E_{\mathrm{m}} = E_{\max} - E_{\min}$. Thus, for even L,

$$\langle n_R\rangle = w - \exp\left[-L(\widetilde{\Delta E\tau})^2 T_R/8\right], \qquad [22]$$

where $w = (2+L)/2$. We note that for odd ring, $w = (L+1)/2$, hence ΔE_{m} is $4 - \pi^2/L^2$. See Figure S7(b). Thus, with L increasing, the broadening of the transition will be narrower, for all the rings with odd or even number of nodes. See Figure S8, where we present numerical confirmation for even rings.

However, if we consider the resonance related to the ground state and the first excited state, for the odd rings, which leads to the transition $w \to w-1$, the L dependence of the energy difference will be distinct. In this case it follows that $\widetilde{\Delta E\tau} = \tau\Delta_1 \mod 2\pi = (E_{1\mathrm{st}} - E_{\mathrm{g}})\tau \mod 2\pi$ with $E_{1\mathrm{st}} - E_{\mathrm{g}} \sim 1/L^2$. i.e. the energy difference shrinks when the system size L grows (see Figure S7(b)). The parameter λ is still proportional to L, and then the term $\lambda(\widetilde{\Delta E\tau})^2$ is proportional to $1/L^3$, when τ is tuned close to the resonance. Hence this will result in an increasing width of the resonance as we increase the size L.

Now it is readily realized that the system size L has various ways of entering the expressions for energy levels and eigenstates. In the context of quantum walks on graphs, this means that the graphs, on which we dispatch quantum walkers, matter. Different graph structures lead to different dispersion relations E_k, as well as the corresponding eigenvectors $|E_k\rangle$. To explore how L determines λ and $\widetilde{\Delta E\tau}$, we checked other graphs.

Complete graph models. One example is the complete graph, in which each vertex is connected to every other vertex. See Figure S6(c). Specifically, the governing Hamiltonian in matrix form, has all elements equal $-\gamma$ except for the diagonal. To achieve a fair comparison, the hopping rate is usually chosen as $\gamma = \gamma_0/L$, and we set $\gamma_0 = 1$ here. There are merely two energy levels, $E_0 = \gamma(1-L)$ and $E_1 = \gamma$, and the eigenstate corresponding to $\gamma(1-L)$, or the ground state is

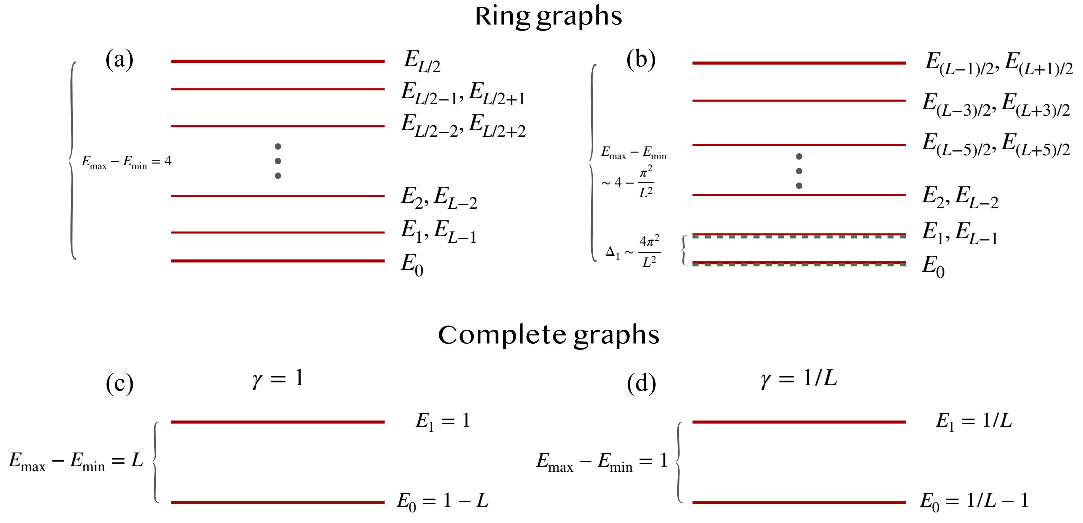

Fig. S7. The energy levels of ring graphs and complete graphs. In (a) we present the case of even L, while in (b) L is odd. We consider the resonance related to the largest energy and the lowest energy (ground state energy), which we called the min-max condition. As a second option we choose the ground state energy and the first excited state energy. The dispersion relation for rings is $E_k = -2\gamma\cos(2\pi k/L)$ with $k = 0, 1, 2, \ldots, L-1$ and $\gamma = 1$. Here γ is the hopping amplitude between nodes, namely H is the adjacency matrix of the graph multiplied by γ. For complete graphs (subplot (c)), the energies are 1 and $1 - L$. As typically used in literature, the hopping rate γ is set as inversely proportional to the number of edges of each vertex, see subplot (d) where the energy difference is $E_{\max} - E_{\min} = 1$.

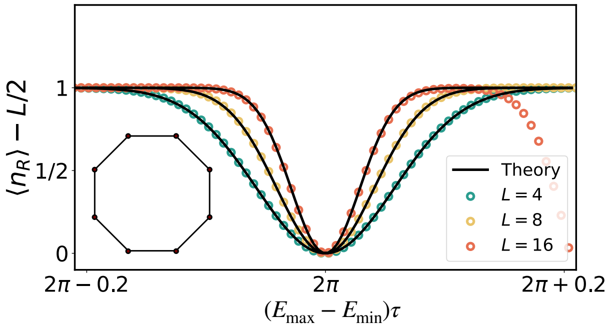

Fig. S8. Restarted mean hitting times versus $(E_{\max} - E_{\min})\tau$, for ring graphs of various sizes L (see inset for an example). The resonances become narrower as we increase the size of the system. Recall, that difference between the largest and ground-state energies, $E_{\max} - E_{\min} = 4\gamma$, is size-independent, and we choose $\gamma = 1$. We shift the mean by $L/2$, to focus on the width of the transition. The numerical results are obtained with equations (2-4) in the main text, and this perfectly matches our theory, see equation (22). The deviation on the right for $L = 16$ is caused by the proximity of another resonance. $T_R = 300$ is used here. Similar results for $\langle n \rangle_{\text{Con}}$ were also tested, and not presented hereinafter.

$(1, 1, 1, \ldots, 1)/\sqrt{L}$, hence the overlaps, for any initial/target state, are $p_+ = 1/L$ and $p_- = (L-1)/L$. This further leads to $\lambda = p_+ p_- / (p_+ + p_-)^3 = (L-1)/L^2 \sim 1/L$ as L is large. Hence with equations (13) and (14) in the text, we have for large L,

$$\langle n_R \rangle \sim w - \exp\Big[-(\widetilde{\Delta E \tau})^2 T_R / L\Big], \qquad [23]$$

where $w = 2$ and $\widetilde{\Delta E \tau} = \tau \Delta E_{\text{m}} \mod 2\pi = L\gamma\tau \mod 2\pi$. From here we see that if choosing γ independent on L, say $\gamma = 1$, we will have $\widetilde{\Delta E \tau} = L\tau \mod 2\pi$ since the energy difference becomes L, as shown in Figure S7(c). Then equation (23) becomes $\langle n_R \rangle \sim w - \exp\big[-L(\tau - 2\pi/L)^2 T_R\big]$ when $\tau \simeq 2\pi/L$, indicating again a decreasing width of resonance as L grows.

As mentioned above, we could also choose $\gamma = 1/L$ as done in the literature of quantum walks, which leads the energy difference to $\Delta E_{\text{m}} = 1$, as shown in Figure S7(d). Then we have $\langle n_R \rangle \sim w - \exp\big[-(\tau \mod 2\pi)^2 T_R / L\big]$, suggesting an increasing width of resonance with the system size L increasing. See Figure S9 for the graphic demonstration.

Linear segments, bipartite graphs. We also checked linear segments, and complete bipartite graphs $K_{L/2,L/2}$ (See Figure S6(c) and (d)), for both of them and around the resonance where $\{e^{-i\tau E_{\max}}, e^{-i\tau E_{\min}}\}$ merge, the prefactor of $(\tau - \tau_c)^2$ (with τ_c the resonance τ) is proportional to $L^3 T_R$, suggesting again narrower broadening as L grows. More concretely, for a line of size L, the Hamiltonian is $H = -\gamma \sum_{x=1}^{L-1} (|x\rangle\langle x+1| + |x+1\rangle\langle x|)$, whose energy levels are $E_k = -2\gamma\cos[k\pi/(L+1)]$ with $k = 1, 2, \ldots, L$, and the corresponding eigenvectors are $|E_k\rangle = \sqrt{2/(L+1)} \sum_{j=1}^{L} \sin[k\pi j/(L+1)]\,|j\rangle$. Hence there are L distinct energies (no degeneracy), with the largest (lowest) energy $E_L = 2\gamma\cos[\pi/(L+1)]$ ($E_1 = -E_L$), and the overlaps, for certain target site x_{d}, are $p_k = |\langle x_{\text{d}}|E_k\rangle|^2 = [2/(L+1)]\sin^2[k\pi x_{\text{d}}/(L+1)]$. Without loss of generality, assuming

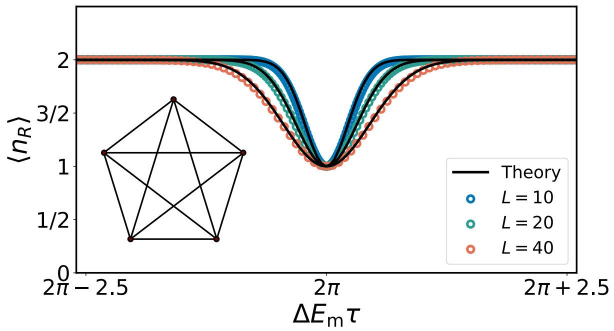

Fig. S9. Restarted mean hitting time for complete graphs of different sizes (see inset for an example). The numerical results are obtained with equations (2-4) in the main text, and the theory is computed with equation (23). Here $\Delta E_{\mathrm{m}} = 1$ and we used $\gamma = 1/L$ for a fair comparison. Unlike Figure S8, the broadening becomes wider as the system size L increases. Here we used $T_R = 100$.

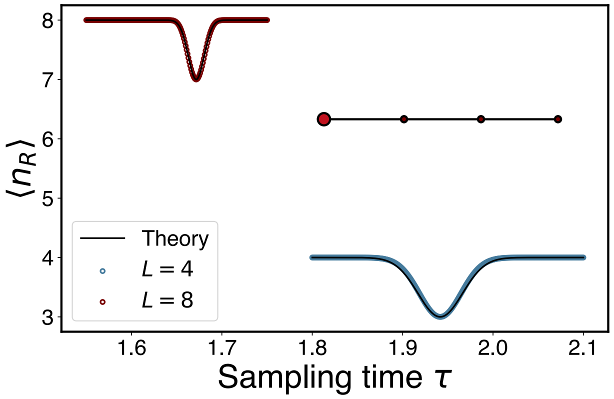

Fig. S10. Restarted mean hitting time for finite segments of different sizes. Repeated measurements are made on the leftmost node (see schematics in the inset, where the larger circle points to the measured node). The numerical results are obtained with equations (2-4) in the main text, and the theory is computed with equation (24). We see that the broadening becomes narrower as the system size L increases. Here $T_R = 100$.

odd L, we consider x_{d} at either end of the line, or the middle of the line, namely, $x_{\mathrm{d}} = 1$ or $x_{\mathrm{d}} = (L+1)/2$, leading to $p_k = |\langle 1|E_k\rangle|^2 = [2/(L+1)]\sin^2[k\pi/(L+1)]$, or $p_k = |\langle \frac{L+1}{2}|E_k\rangle|^2 = [2/(L+1)]\sin^2(k\pi/2) = [1-(-1)^k]/(L+1)$, respectively. We find that the p_k's are non-zero for the former case, while for the latter, $x_{\mathrm{d}} = (L+1)/2$, there appear $p_l = 0$ when l is even. This leads to different winding numbers for the two cases, since w is equal to the number of distinct phases $e^{-iE_k\tau}$ associated with non-zero p_k. Hence $w = L$ for the case $x_{\mathrm{d}} = 1$, and $w = (L+1)/2$ for the case $x_{\mathrm{d}} = (L+1)/2$. We now focus on the resonance where phases $\{e^{-i\tau E_1}, e^{-i\tau E_L}\}$ merge at $\tau_c = 2\pi/|E_1 - E_L| = \pi/2\gamma\cos[\pi/(L+1)]$, which is the smallest resonance τ except for $\tau = 0$. For the target at one end of the line, $x_{\mathrm{d}} = 1$, the corresponding overlaps to E_1 and E_L are $p_1 = p_L = [2/(L+1)]\sin^2[\pi/(L+1)] \approx 2\pi^2/(L+1)^3$, with the approximation valid when L is large. For the case $x_{\mathrm{d}} = (L+1)/2$, we have $p_1 = p_L = 2/(L+1)$. Therefore, for $x_{\mathrm{d}} = 1$, namely the end node on the segment, equations (13) and (14) for large L become

$$\langle n_R\rangle = w - \exp\left\{-\frac{(L+1)^3}{16\pi^2}\left[4\cos\left(\frac{\pi}{L+1}\right)\tau \mod 2\pi\right]^2 T_R\right\}, \quad [24]$$

where $w = L$. And for the target site at the middle of the line, $x_{\mathrm{d}} = (L+1)/2$, we have

$$\langle n_R\rangle = w - \exp\left\{-\frac{L+1}{16}\left[4\cos\left(\frac{\pi}{L+1}\right)\tau \mod 2\pi\right]^2 T_R\right\}, \quad [25]$$

where $w = (L+1)/2$. Clearly, these expressions exhibit a different dependence on system size. See Figures S10 and S11 for numerical confirmation, where the theory works well and predicts the narrowing of broadening of resonances as the system becomes larger.

Another example is a complete bipartite graph, also called a complete bi-colored graph, usually denoted by $K_{l,m}$, see Figure S6(d). The vertices of the graph can be decomposed into two disjoint sets, containing l and m elements, respectively, such that *no* two vertices within the *same* set are connected by an edge, and every pair of vertices from *different* sets are connected. See Figure S6(d) for schematics of $K_{3,3}$. Here we use $K_{L/2,L/2}$ to demonstrate the influence of size L on the restart uncertainty relation. The Hamiltonian governing a quantum walk on such a graph is $H = -\gamma\begin{bmatrix} O & C \\ C & O\end{bmatrix}$ with C a $\frac{L}{2}\times\frac{L}{2}$ matrix with

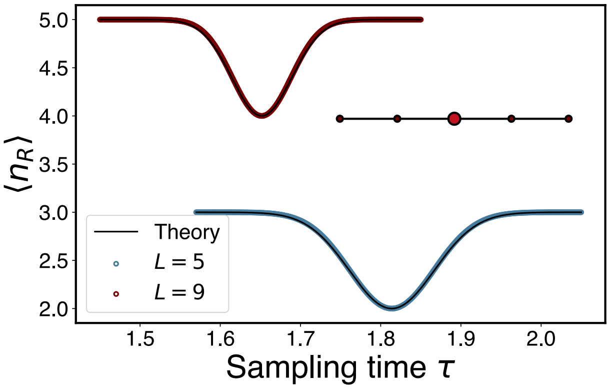

Fig. S11. Restarted mean hitting time for finite segments of different sizes, here the target is set at $x_{\mathrm{d}} = (L+1)/2$. The numerical results are obtained with equations (2-4) in the main text, and the theory is computed with equation (25). We see that the broadening becomes narrower as the system size L increases. $T_R = 40$ is used. Here measurement is performed on the middle node, see inset.

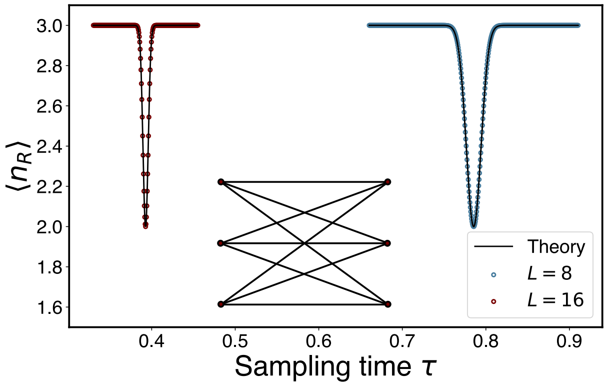

Fig. S12. Restarted mean hitting time for complete bipartite graphs $K_{L/2,L/2}$ with different L (see an example in Figure S6d). The numerical results are obtained with equations (2-4) in the main text, and the theory is computed with equation (27). We see that the broadening becomes narrower as the system size L increases. Here $T_R = 100$.

all elements as 1. The energy levels are $\gamma\{-L/2, 0, L/2\}$. The eigenvectors corresponding to the lowest and largest energies are $|E_0\rangle = (-1,-1,-1,\cdots,-1,-1,-1,\cdots)^T/\sqrt{L}$, and $|E_2\rangle = (-1,-1,-1,\cdots,1,1,1,\cdots)^T/\sqrt{L}$. This gives, around the resonance where $\{e^{-i\tau E_0}, e^{-i\tau E_2}\}$ merge, the overlaps $p_0 = p_2 = 1/L$, for any node as the target site. Hence the parameters are straightforwardly calculated, namely $\lambda = L/8$, and $\widetilde{\Delta E\tau} = \tau L \mod 2\pi$ (γ is set as 1). Thus the statistical measures of mean hitting time around the resonance is

$$\langle n_R \rangle = w - \exp\left[-L(\widetilde{\Delta E\tau})^2 T_R/8\right], \qquad [26]$$

where $w = 3$. In the vicinity of the resonance $\widetilde{\Delta E\tau} \simeq 0$, $(\tau L \mod 2\pi)^2$ becomes $(\tau L - 2\pi)^2$, thus, we get

$$\langle n_R \rangle = w - \exp\left[-L^3(\tau - 2\pi/L)^2 T_R/8\right], \qquad [27]$$

In Figure S12 we present the numerical results calculated with equations (2-4) in the main text, and our theory agrees excellently with the numerics. Therefore, as theoretically predicted and numerically seen, the increasing system size leads to more abrupt transitions of the mean hitting times, namely the resonance is narrowed as we increase L.

4. Effects of non-precise sampling time and restart time

We elaborate here how we implement the randomness of τ and T_R in our problem, and what we witness for their effects upon the restart uncertainty relation and the broadening of resonance phenomenon.

A. Randomness in the sampling time τ. For the randomness in the evolution time τ, we employed the Monte Carlo method to explore the impact of fluctuations of τ on the uncertainty relation. We denote the actual evolution time in experiments by $\tilde{\tau}$, and it is a uniformly random variable within the range $[\tau(1-\nu), \tau(1+\nu)]$. We will vary the value of ν from 0.05 to 0.3

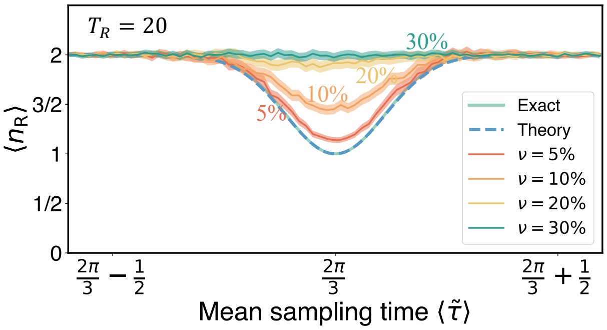

Fig. S13. Mean hitting time versus the mean sampling time $\langle \tilde{\tau} \rangle$, for the three-site ring model with varying inaccuracy levels in the evolution time τ and fixed $T_R = 20$. Utilizing the Monte Carlo method with $30,000$ realizations (conducted with *Python*), we find that as the fluctuations of τ increase, the resonances are progressively diminished, yet the topological number $\langle n_R \rangle = 2$, far from the resonance, remains unaffected and exhibits robustness.

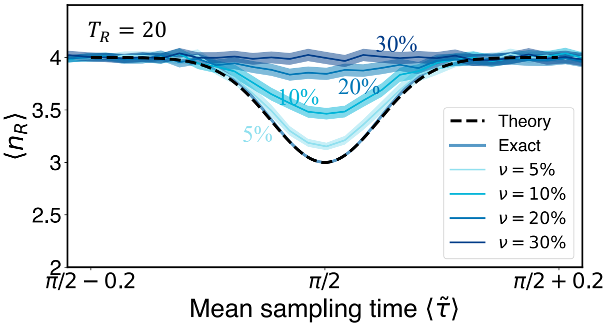

Fig. S14. Mean hitting time versus the mean sampling time $\langle \tilde{\tau} \rangle$, for the benzene-type ring model, with varying inaccuracy levels in the evolution time τ and a fixed restart time $T_R = 20$. Utilizing the Monte Carlo method, we simulated the first detection process with restarts across $30,000$ realizations. Our results demonstrate that as the fluctuations of τ increase, the resonances are progressively diminished, yet the topological number $\langle n_R \rangle = 4$ remains unaffected and exhibits robustness.

corresponding to 5% to 30% inaccuracy levels. Here we use the three-site ring model, which was used in our IBM experiment. We also study the benzene ring model. See Figure S13-S15 for numerical results,

The procedures for the Monte Carlo method, used to produce Figures S13,S14,S15, are the following:

(i) *Initialization of the quantum walker:* The quantum walker is initially evolved from a predefined state in accordance with the Schrödinger equation This evolution occurs over a time duration, $\tilde{\tau}_1$, which is a uniformly random variable within the range $[\tau(1-\nu), \tau(1+\nu)]$.

(ii) *Random coin tossing for detection assessment:* A random variable, referred to as a "coin", is generated. This variable is uniformly distributed within the interval $[0, 1]$. The purpose of the coin is to ascertain whether the quantum walker is detected following the initial state's evolution. This determination is made by comparing the coin's value with the detection probability, which is derived from the unitary evolution.

(iii) *Non-detection and state modification:* If the coin value falls below the computed detection probability, we are done and the hitting time is 1. If the coin value exceeds the computed detection probability, it signifies that the walker remains undetected. In this case, the amplitude at the target site $|0\rangle$ is erased, and the wave vector is renormalized. Subsequently, the single-site-erased wave vector undergoes unitary evolution for a duration, $\tilde{\tau}_2$. Notably, $\tilde{\tau}_2$ is an independent and identically distributed (i.i.d.) random value, akin to $\tilde{\tau}_1$. The objective is to compute the probability of detection at the time $t = \tilde{\tau}_1 + \tilde{\tau}_2$.

(iv) *Repeated detection attempts:* Post the initial non-detection, a second i.i.d. coin is generated and compared with the newly computed detection probability to decide if the walker is detected at this stage, as in the step (iii).

(v) *Criteria for repetition termination under sharp restart:* The process iterates until the coin value is less than the computed probability of detection, marking the end of a repetition cycle. Alternatively, if the process extends up to a preset fixed restart step, T_R (i.e. after a cumulative time of $t = \tilde{\tau}_1 + \tilde{\tau}_2 + \cdots + \tilde{\tau}_{T_R}$), and the walker remains undetected, the entire procedure recommences from the initial state, repeating the procedures (i)-(v).

(vi) *Conditional/restarted hitting time calculation:* Once the system is detected in the target state, for the first time, we are done. The number of all preceding unsuccessful attempts, plus 1, is recorded as the fist-detection time, or the hitting

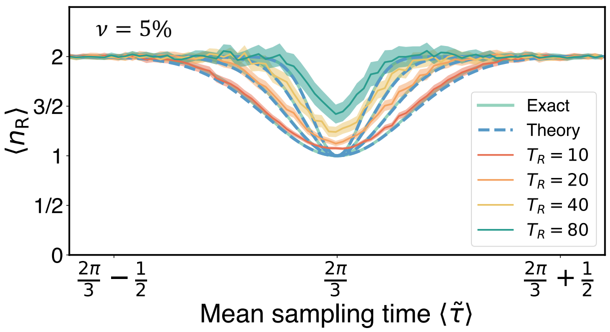

Fig. S15. Mean hitting time versus the mean sampling time $\langle\tilde{\tau}\rangle$, for the three-site ring model, with a fixed inaccuracy level of 5% in the evolution time τ, and varying restart time from $T_R = 10$ to $T_R = 80$. Using Monte Carlo simulation with $30,000$ realizations, we observed that deviations in the resonances intensify with increasing restart time T_R. However, despite these deviations, when $\langle\tilde{\tau}\rangle$ is either small or large, namely when $\langle\tilde{\tau}\rangle$ is tuned far from the resonance, we see that randomness of τ is of no consequence, and the topological winding number 2, is robust.

time under restarts, n_R. For the conditional mean, we need to discard all data where no detection occurs before each restart, namely, only the outcome sequences containing "yes" are retained (as explained in the main text).

(vii) *Realizations and expected value determination:* The aforementioned procedures, executed for obtaining a single value of the hitting time under T_R-step restarts, is called a single realization. To ascertain $\langle n\rangle_{\text{Con}}$ or $\langle n_R\rangle$ as a function of τ, large number of realizations are conducted for each value of τ.

Our results indicate that at $\nu = 5\%$, the uncertainty relation exhibits minimal change in the mean recurrence time, as demonstrated in Figure S13, and only a slight deviation in the mean. As the fluctuations of τ increase, these deviations become progressively more pronounced. Notably, at an inaccuracy level of 30%, the resonances are completely obliterated, effectively disrupting the uncertainty relations due to the stochastic nature of the evolution time, τ. Furthermore, our analysis reveals that such fluctuations of τ does not affect the topological number, which is 2 in this case, underscoring its robustness. This resilience may represent a form of topological self-protection. A similar phenomenon has also been observed in the benzene-type ring model, as illustrated in Figure S14.

We further investigate the scenario with a constant inaccuracy level of 5% and a variable restart time, T_R, to examine the influence of increasing T_R on the system dynamics, as illustrated in Figure S15. Our observations reveal that the deviation from the results for ideal cases (without noise) increases when T_R grows. Moreover, at the exact point $\tau = 2\pi/3$, the mean hitting times depart from $w = 1$ of the precise-τ process. Despite these changes, the topological number far from the resonance remains stable, underscoring its robustness against variations in the sampling time.

B. Randomness in the restart time T_R. Recall that previously, we recorded T_R times, which is the duration of the experiment in units of τ. Clearly, in common situations with mid-circuit measurements on quantum computers, this number is fixed since experimentalists can typically control and measure the duration of an experiment. But in the literature of stochastic restarts the randomness of this variable is also considered for classical restart processes. We will now investigate how the randomness in T_R affects our uncertainty relation. We assume the restart time T_R assigned to three values, 19, 20 and 21, with probability $1/4$, $1/2$ and $1/4$, respectively (the mean of T_R is still 20, motivated by our quantum computer experiments). We computed both exact numerical results (see the formulas below), and simulated results with Monte Carlo methods, as shown in Figure S16. Our analysis reveals that, in each case, the randomness in T_R exerts negligible impact on both the uncertainty relations and the stability of the topological number.

Our initial choice of distribution of T_R was rather narrow, we therefore also studied the case when T_R is Poisson distributed. We have found that also in this case, the effect of randomness in T_R is negligible. The reason is the following: the mean of T_R was 20, similar to our IBM experiments. In this case, the Poisson distribution is roughly symmetric around its mean, similar to a normal distribution. The important issue is that when T_R is fixed, the location of the resonance τ is independent of T_R and further, the width of the resonance is inversely proportional to T_R. Hence, we expect that for a distribution of T_R symmetric around the mean (again, like the normal distribution or a tent distribution), the effects of randomness of T_R are negligible. For non-symmetric distributions of T_R, other effects are expected.

Therefore, to summarize, for symmetric distributions of T_R, the peak of the distribution is located on the mean, the time-energy uncertainty relation does not change considerably if compared with a theory for which T_R is fixed. And for fluctuations of the sampling time τ, our analysis reveals that, when T_R is not too large, the time-energy uncertainty relation is not significantly affected. But the resonance is diminishing when T_R is increased for fixed width of the distribution of τ. At the same time, the topological number far from resonance is very robust to the fluctuations of τ.

Formulas for random restart time. The numerical results for random T_R are calculated using the following formula:

$$\langle n_R\rangle = \frac{\sum_{k=1}^{\infty} kP(T_R = k)(1 - \sum_{n=1}^{k-1} F_n) + \sum_{n=1}^{\infty} nF_n \sum_{k=n+1}^{\infty} P(T_R = k)}{\sum_{n=1}^{\infty} F_n \sum_{k=n}^{\infty} P(T_R = k)}. \quad [28]$$

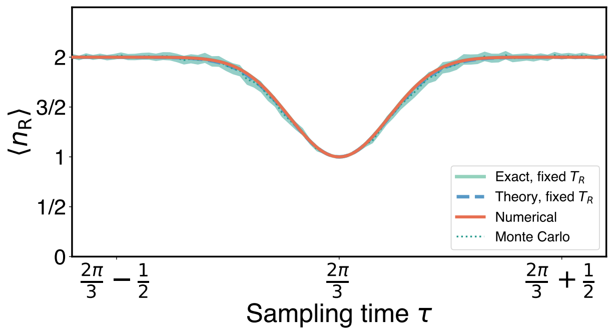

Fig. S16. Mean hitting time for the three-site ring model with random T_R, where T_R is drawn from a narrow distribution, such that the probabilities of T_R being 19, 20, and 21 are $1/4$, $1/2$, and $1/4$, respectively. Here we used the mean of T_R equal to 20, motivated by our quantum computer experiments. Our findings reveal that this randomness in T_R has a negligible impact on the outcomes. Exact results for the restarted mean with fixed T_R are obtained with equations (2-4) in the main text, numerical results are calculated with equation (30), and the Monte Carlo simulations are conducted with *Python*.

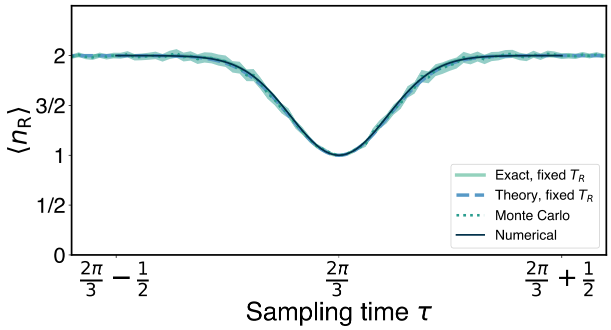

Fig. S17. Mean hitting time for the three-site ring model with Poissonian distributed T_R, and we used $\langle T_R \rangle = 20$. There appears no deviation from the fixed-T_R case, for which the exact results are obtained with equations (2-4) in the main text, and the theory is computed with equation (7) in the main text. The numerical results for Poissonian distributed T_R are calculated with equation (31) below, and the Monte Carlo simulations are conducted with $30,000$ realizations.

$P(T_R = k)$ is the probability that restart occurs after k attempts of measurements, and F_n is the probability of detecting the system at the nth measurement for the first time, in the absence of restarts. These basic probabilities are found using equation (2) in the main text. In equation (28), we employed the general framework proposed by Pal and Reuveni (5, 6), which states that the mean hitting time with a general distribution of T_R is

$$\langle n_R \rangle = \frac{\langle \min(n, T_R) \rangle}{P(n \leq T_R)}, \qquad [29]$$

where n is the first hitting time in the absence of restart, and the numerator means the expectation of the minimum of n and the random restart time T_R. We note that

$$\begin{aligned} \langle \min(n, T_R) \rangle &= \sum_{k=1}^{\infty} k P(T_R = k) \left(1 - \sum_{n=1}^{k-1} F_n \right) + \sum_{n=1}^{\infty} n F_n \sum_{k=n+1}^{\infty} P(T_R = k), \\ P(n \leq T_R) &= \sum_{n=1}^{\infty} F_n \sum_{k=n}^{\infty} P(T_R = k). \end{aligned} \qquad [30]$$

Here we used the normalization of $P(T_R = k)$, i.e. $\sum_{k=1}^{\infty} P(T_R = k) = 1$. And for the aforementioned distributions of T_R on a finite range, the upper limit of the sum associated with $P(T_R = k)$ will be truncated to the largest value of T_R. For the Poisson distribution of T_R, equation (28) becomes (6, 7)

$$\langle n_R \rangle_{\text{Pois}} = \frac{1 + \lambda - \sum_{n=1}^{\infty} F_n \sum_{k=n+1}^{\infty} (k-n) \frac{e^{-\lambda} \lambda^{k-1}}{(k-1)!}}{\sum_{n=1}^{\infty} F_n \sum_{k=n}^{\infty} \frac{e^{-\lambda} \lambda^{k-1}}{(k-1)!}}, \qquad [31]$$

Here the parameter $\lambda = \langle T_R \rangle - 1$. In Figures S16,S17, we utilized equation (31) for the Poisson case, and (28) for the tent-like distribution of T_R, to generate the “Numerical” results.

5. Implementation on a quantum computer

The three-site tight-binding Hamiltonian (equation (16) in the main text with $L = 3$) is encoded by the qubit Hamiltonian:

$$H = -\frac{1}{2}(\sigma_{x,1} + \sigma_{x,2} + \sigma_{z,1}\sigma_{x,2} + \sigma_{x,1}\sigma_{z,2} + \sigma_{x,1}\sigma_{x,2} + \sigma_{y,1}\sigma_{y,2})$$

where σ_x, σ_y and σ_z are the Pauli matrices. The Hamiltonian H defines two disconnected subspaces, the first composed of the states $|00\rangle, |01\rangle, |10\rangle$ and the second from $|11\rangle$. In our scheme, the state $|11\rangle$ is not used. Hence, we use the following mapping between the qubit and spatial states representation: $|01\rangle \to |0\rangle, |10\rangle \to |2\rangle$ and $|00\rangle \to |1\rangle$. The unitary evolution operator $U(\tau) = \exp(-iH\tau)$, must be constructed on a quantum computer as a product of elementary gate operators. We explain how to perform the measurements and how to construct an efficient unitary.

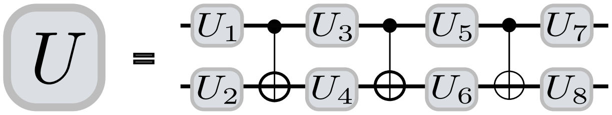

Fig. S18. Decomposition of the Unitary

We define the two-qubit unitary transformation $U(\tau) = \exp(-iH\tau)$ using Cartan's decomposition (8), namely with three CNOT gates and single unitary gates (see sketch). Importantly, this allows us to vary τ in simulations without much computational cost. For larger systems, one would have to use other methods to model the unitary, namely trotterization technique.

In our study, we employ localized single-site measurements, as integrated in the IBM computer toolbox, to detect state $|0\rangle$ without distinguishing states $|1\rangle$ and $|2\rangle$, as mentioned in the main text. As an error suppression strategy, we are using dynamical decoupling and inserting two XX-gates on the qubit which is not measured to keep it coherent. The schematic timeline is given by Fig. S19.

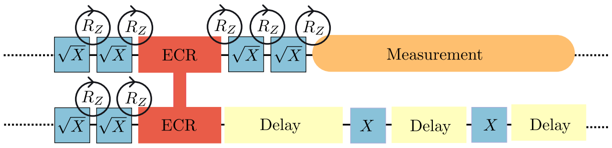

Fig. S19. The schematic timeline for qubit gates in the quantum computer

References

1. IBM Noise Models (https://docs.quantum.ibm.com/guides/build-noise-models) (2024) Accessed: 2024-08-08.
2. H Friedman, DA Kessler, E Barkai, Quantum walks: The first detected passage time problem. *Phys. Rev. E* **95**, 032141 (2017).
3. FA Grünbaum, L Velázquez, AH Werner, RF Werner, Recurrence for Discrete Time Unitary Evolutions. *Commun. Math. Phys.* **320**, 543–569 (2013).
4. F Thiel, I Mualem, D Meidan, E Barkai, DA Kessler, Dark states of quantum search cause imperfect detection. *Phys. Rev. Res.* **2**, 043107 (2020).
5. A Pal, S Reuveni, First passage under restart. *Phys. Rev. Lett.* **118**, 030603 (2017).
6. OL Bonomo, A Pal, First passage under restart for discrete space and time: Application to one-dimensional confined lattice random walks. *Phys. Rev. E* **103**, 052129 (2021).
7. R Yin, E Barkai, Restart expedites quantum walk hitting times. *Phys. Rev. Lett.* **130**, 050802 (2023).
8. G Vidal, CM Dawson, Universal quantum circuit for two-qubit transformations with three controlled-not gates. *Phys. Rev. A* **69**, 010301 (2004).
